# Disruption of the ATXN1-CIC complex reveals the role of additional nuclear ATXN1 interactors in spinocerebellar ataxia type 1

**DOI:** 10.1016/j.neuron.2022.11.016

**Published:** 2022-12-27

**Authors:** Stephanie L. Coffin, Mark A. Durham, Larissa Nitschke, Eder Xhako, Amanda M. Brown, Jean-Pierre Revelli, Esmeralda Villavicencio Gonzalez, Tao Lin, Hillary P. Handler, Yanwan Dai, Alexander J. Trostle, Ying-Wooi Wan, Zhandong Liu, Roy V. Sillitoe, Harry T. Orr, Huda Y. Zoghbi

**Affiliations:** 1 Program in Genetics and Genomics, Baylor College of Medicine, Houston, TX 77030, USA; 2 Department of Molecular and Human Genetics, Baylor College of Medicine, Houston, TX 77030, USA; 3 Jan and Dan Duncan Neurological Research Institute at Texas Children’s Hospital, Houston, TX 77030, USA; 4 Program in Developmental Biology, Baylor College of Medicine, Houston, TX 77030, USA; 5 Medical Scientist Training Program, Baylor College of Medicine, Houston, TX 77030, USA; 6 Program in Integrative Molecular and Biomedical Sciences, Baylor College of Medicine, Houston, TX 77030, USA; 7 Department of Pathology & Immunology, Baylor College of Medicine, Houston, TX 77030, USA; 8 Department of Laboratory Medicine and Pathology, Institute for Translational Neuroscience, University of Minnesota, Minneapolis, MN 55455, USA; 9 Department of Pediatrics, Baylor College of Medicine, Houston, TX 77030, USA; 10 Department of Neuroscience, Baylor College of Medicine, Houston, TX 77030, USA; 11 Development, Disease Models & Therapeutics Graduate Program, Baylor College of Medicine, Houston, TX 77030, USA; 12 Howard Hughes Medical Institute, Baylor College of Medicine, Houston, TX 77030, USA; 13 Lead contact

## Abstract

Spinocerebellar ataxia type 1 (SCA1) is a paradigmatic neurodegenerative disease in that it is caused by a mutation in a broadly expressed protein, ATXN1; however, only select populations of cells degenerate. The interaction of polyglutamine-expanded ATXN1 with the transcriptional repressor CIC drives cerebellar Purkinje cell pathogenesis; however, the importance of this interaction in other vulnerable cells remains unknown. Here, we mutated the 154Q knockin allele of *Atxn1*^*154Q/2Q*^ mice to prevent the ATXN1-CIC interaction globally. This normalized genome-wide CIC binding; however, it only partially corrected transcriptional and behavioral phenotypes, suggesting the involvement of additional factors in disease pathogenesis. Using unbiased proteomics, we identified three ATXN1-interacting transcription factors: RFX1, ZBTB5, and ZKSCAN1. We observed altered expression of RFX1 and ZKSCAN1 target genes in SCA1 mice and patient-derived iNeurons, highlighting their potential contributions to disease. Together, these data underscore the complexity of mechanisms driving cellular vulnerability in SCA1.

## In brief

Coffin et al. show that global ablation of the ATXN1-CIC interaction normalizes aberrant SCA1 CIC genome-wide binding in mice; however, it does not correct all transcriptional or behavioral changes. They discovered that ATXN1 interacts with multiple factors to drive SCA1, revealing the mechanistic complexity that underlies regional vulnerability in neurodegenerative diseases.

## Graphical Abstract

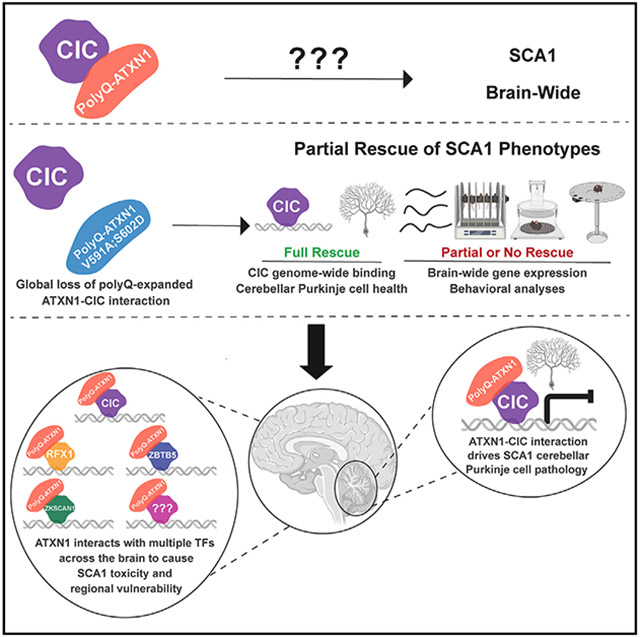

## INTRODUCTION

Several neurodegenerative disorders, such as Alzheimer’s and Parkinson’s disease, are characterized by region and cell-specific degeneration, despite broad expression of the disease-causing protein.^1^ However, the mechanisms driving this regional vulnerability are unknown. One such disease, spinocerebellar ataxia type 1 (SCA1), is an autosomal dominant neurodegenerative disorder affecting 1 in 100,000 people.^2^ Symptoms of SCA1 include motor incoordination, dysarthria, and breathing and swallowing deficits due to degeneration of cerebellar and brainstem neurons.^3^ SCA1 is caused by the expansion of CAG repeats encoding the polyglutamine (polyQ) tract in ATAXIN-1 (ATXN1).^4,5^

ATXN1 forms a native complex with the transcriptional repressor Capicua (CIC) and when polyQ-expanded ATXN1 binds CIC, the complex acquires a toxic gain of function that results in the hyper-repression of CIC target genes.^6,7^ A transgenic mouse model expressing polyQ-expanded *ATXN1* exclusively in cerebellar Purkinje cells (PCs) tested the importance of the ATXN1-CIC complex in SCA1 cerebellar phenotypes.^8^ Prevention of ATXN1-CIC binding rescued SCA1 cerebellar phenotypes,^9^ demonstrating that this interaction is critical for cerebellar PC pathology. A key question, however, is whether CIC mediates ATXN1 toxicity in other vulnerable cells in SCA1.

Here, we sought to resolve the brain-wide contributions of the ATXN1-CIC complex in SCA1 by mutating the two amino acids critical for the interaction of ATXN1 with CIC in a SCA1 knockin mouse model (*Atxn1*^*154Q/2Q*^) that recapitulates all human SCA1 phenotypes.^10^ Surprisingly, disruption of the ATXN1-CIC complex only partially improved motor coordination, respiration, and lifespan but did not affect other phenotypes. With these findings, it was evident that CIC is not the sole driver of SCA1 toxicity; hence, we conducted molecular and proteomic analyses to reveal CIC-independent molecular changes and searched for novel drivers of disease. This revealed three novel transcription factors that interact with ATXN1 and could contribute to SCA1 pathogenesis.

## RESULTS

### The ATXN1-CIC interaction is critical for SCA1 pathogenesis in cerebellar purkinje cells

**Figure 1.**
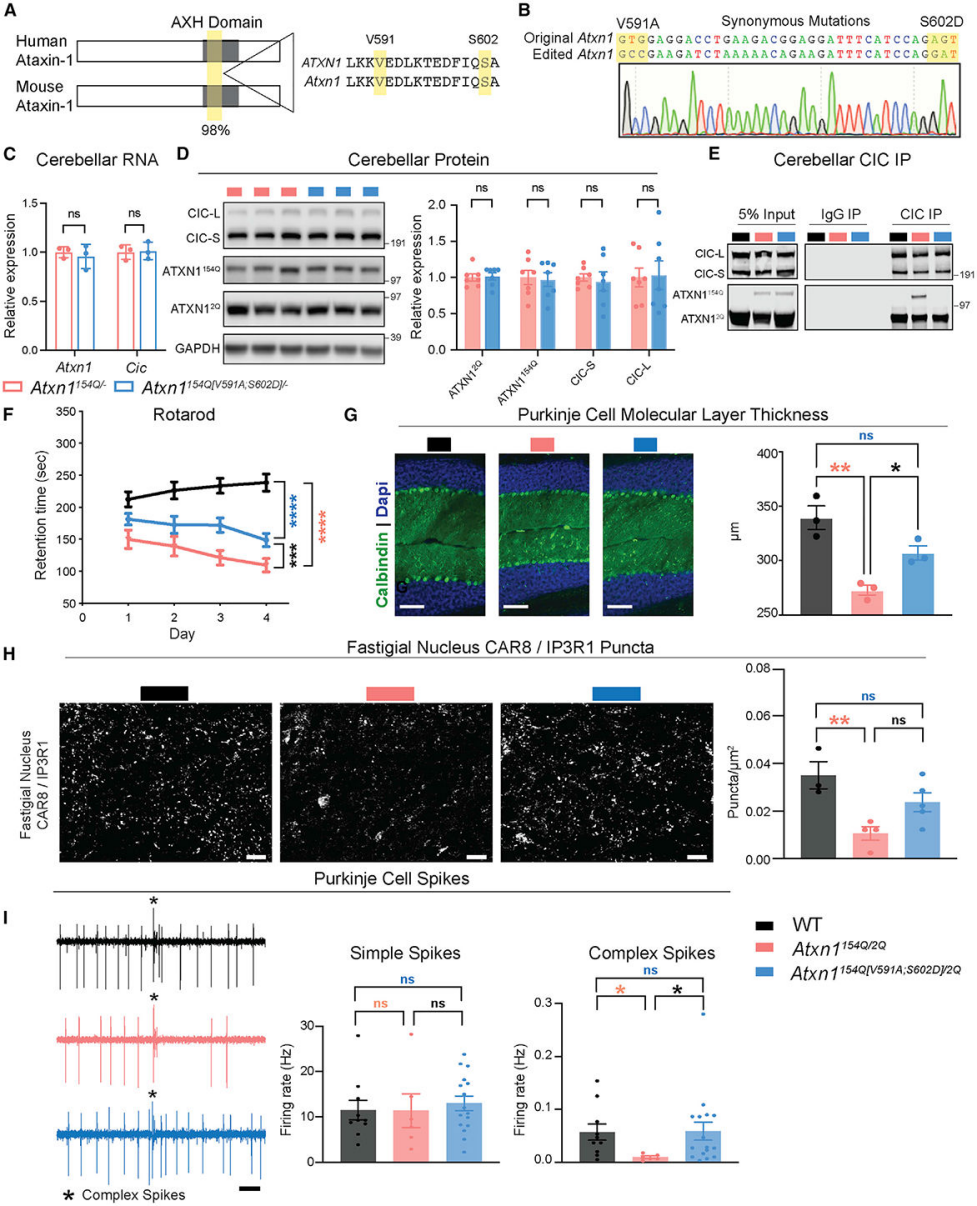
ATXN1-CIC complex is critical for SCA1 pathogenesis in cerebellar Purkinje cells (A) Conservation of ATXN1 AXH domain and amino acids V591 and S602 in human and mouse. (B) Sanger sequencing confirming the correct mutation of V591 and S602 and synonymous mutations in *Atxn1*^*154Q[V591A;S602D]/2Q*^ F1 offspring. (C) Quantification of *Atxn1* and *Cic* RNA levels in the cerebellum of mice at 4 weeks of age. *Atxn1* and *Cic* were normalized to *Gapdh,* n = 3. (D) Representative western blot and quantification of ATXN1 and CIC protein levels in the cerebellum of mice at 4 weeks of age. ATXN1 and CIC were normalized to GAPDH, n = 7. (E) Representative western blot showing the pull-down of ATXN1 and CIC upon immunoprecipitation (IP) of CIC in cerebella of mice at 4 weeks of age. (F) Rotarod assay in mice at 24 weeks of age. n = 9–14. (G) Cerebellar Purkinje cells stained with DAPI and Calbindin at 40 weeks of age (original magnification ×20, scale bars, 100 μm) and quantification of the molecular layer thickness of the cerebellum in lobules V and VI, n = 3. (H) Representative images of CAR8/IP3R1 expression in the fastigial nucleus (scale bars, 20 μm) and quantification of CAR8/IP3R1 puncta, n = 3–5. (I) 1 s example recordings of Purkinje neuron firing. Scale bars, 0.1 s. Complex spike, * (left). Firing rate of Purkinje neurons simple spikes (middle) and complex spikes (right). n = 3, number of cells (c) = 6–16. t tests were used for (C) and (D); two-way ANOVA with Tukey’s multiple comparisons was used for (F); one-way ANOVAs with Tukey’s multiple comparisons were used for (G), (H), and (I). In each case, *, **, ***, ****, and ns denote p < 0.05, p < 0.01, p < 0.001, p < 0.0001, and p > 0.05, respectively. All data are represented as mean ± SEM. See also Figure S1.

Previous work showed that amino acids V591 and S602 in the AXH domain of human ATXN1 are critical for its interaction with CIC and for driving toxicity in cerebellar PCs;^9,11^ however, it remained unclear if the same amino acids regulate the interaction in the mouse and contribute to SCA1 pathology beyond PCs. The AXH domain is 98% conserved between human and mouse, and V591 and S602 exist in mouse (Figure 1A). To test if the function of these amino acids is conserved, we introduced a valine-to-alanine mutation at position 591 (V591A) and a serine-to-aspartic acid mutation at position 602 (S602D) on the expanded *Atxn1*^*154Q*^ allele of the *Atxn1*^*154Q/2Q*^ knockin mouse to generate the novel *Atxn1*^*154Q[V591A;S602D]/2Q*^ model. Sequencing confirmed the correct mutations in the F1 offspring of the CRISPR-Cas9 modified founder mouse (Figure 1B). We found that these mutations did not impact mRNA or protein stability of *Atxn1* and *Cic* (Figures 1C and 1D). Finally, to ensure these mutations do not cause unexpected effects independent of the polyQ-expanded allele, we generated and characterized *Atxn1*^*2Q[V591A;S602D]/2Q*^ mice, which appeared no different from wild-type (WT) animals (Figures S1A-S1D).

To confirm disruption of the ATXN1-CIC interaction in mouse tissue, we immunoprecipitated CIC and blotted for ATXN1. Indeed, the V591A and S602D mutations ablate ATXN1^154Q[V591A;S602D]^ binding to CIC in all brain regions assayed (Figures 1E and S1E). Next, we characterized the motor incoordination phenotype in the SCA1 mice by quantifying their performance on a rotating rod, which we predicted would be rescued in the *Atxn1*^*154Q[V591A;S602D]/2Q*^. mice, similar to the results seen in the PC-specific manipulation.^9^ Surprisingly, the *Atxn1*^*154Q[V591A;S602D]/2Q*^ mice performed similarly to the *Atxn1*^*154Q/2Q*^ mice until 24 weeks of age, when the *Atxn1*^*154Q[V591A;S602D]/2Q*^ mice performed significantly better than the *Atxn1*^*154Q/2Q*^ mice (Figures 1F and S1F-G). The varying degree of behavioral rescue here and in the PC-specific model suggests there are cell-type-specific effects of the ATXN1-CIC complex, which would not be captured in the PC-specific model. To explore the role of the ATXN1-CIC complex, specifically in cerebellar PCs, we first assessed the morphology of PCs. We found that the reduced molecular layer thickness of PCs in *Atxn1*^*154Q/2Q*^ mice, a hallmark of significant dendritic loss,^10^ was rescued in *Atxn1*^*154Q[V591A;S602D]/2Q*^ mice (Figure 1G). Examination of PC terminals in the fastigial nucleus, a key region for mediating vermis PC output to the brain and important in ataxia, demonstrated a reduction in PC terminals in *Atxn1*^*154Q/2Q*^ mice, which was rescued in the *Atxn1*^*154Q[V591A;S602D]/2Q*^ mice (Figure 1H). Last, as a functional measure of PC health, we conducted *in vivo* electrophysiology in anesthetized mice and recorded from PC neurons (Figure 1I). This analysis demonstrated no change in simple spike firing rates between genotypes; however, we identified a significant reduction in complex spike firing rates in *Atxn1*^*154Q/2Q*^ mice, which was corrected to wild-type levels in the *Atxn1*^*154Q[V591A;S602D]/2Q*^ mice (Figure 1I). The preservation of simple spike rate indicates that polyQ-expanded ATXN1 does not grossly alter the intrinsic properties of PC function, although the degeneration of PC dendrites (Figure 1G) impacts their functional innervation by climbing fibers, as measured by abnormal complex spike activity.

These data show that V591A and S602D mutations fully ablate the ATXN1-CIC interaction brain-wide, and although this complex is key for SCA1 PC pathology, it is not the sole driver of toxicity in all cell types, as motor incoordination is only partially improved in *Atxn1*^*154Q[V591A;S602D]/2Q*^ mice.

### Loss of ATXN1-CIC binding partially improves SCA1 neurological phenotypes brain-wide

**Figure 2.**
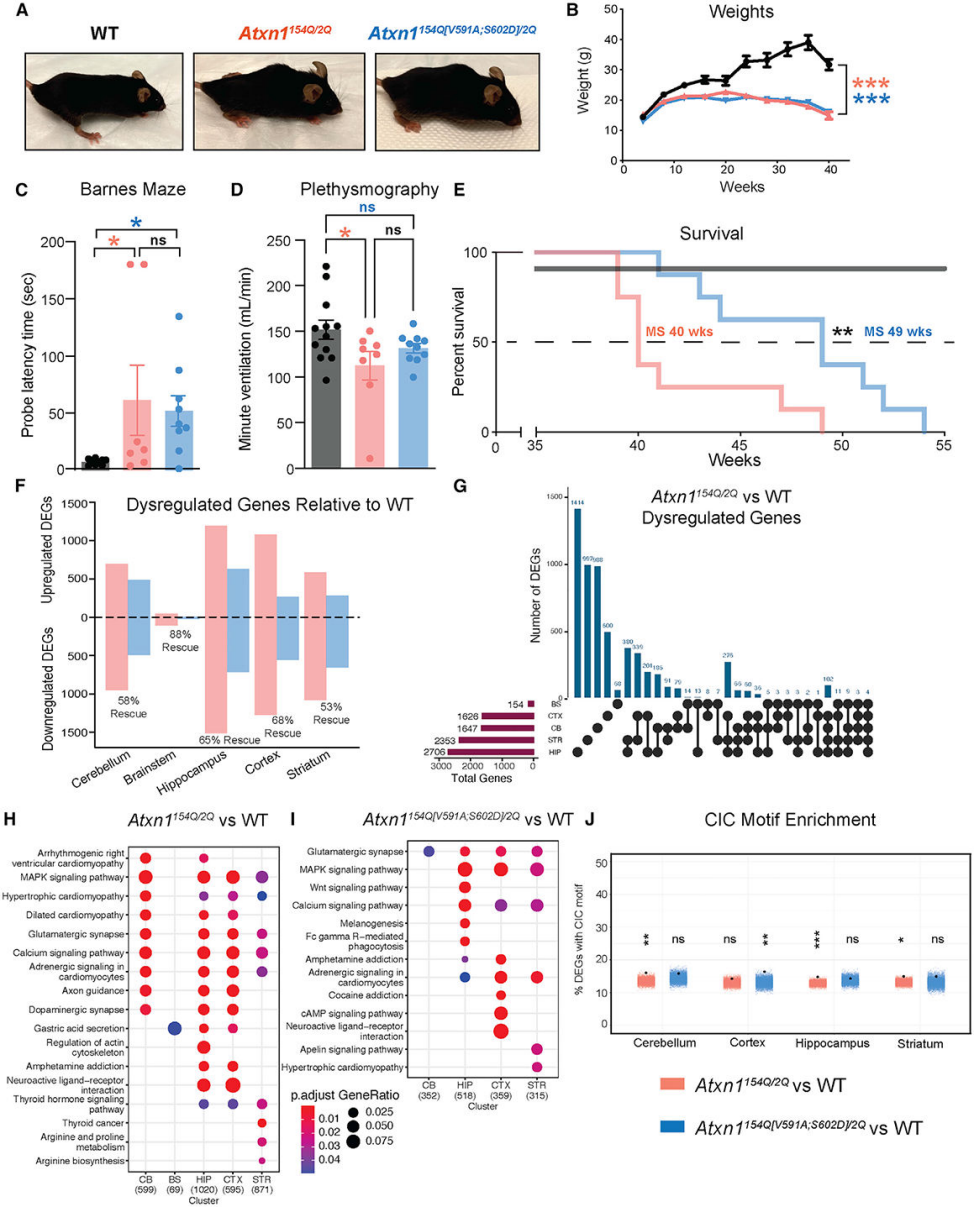
Global loss of the ATXN1^154Q^-CIC interaction partially improves some SCA1 neurological phenotypes (A) *Atxn1*^*154Q/2Q*^ and *Atxn1*^*154Q[V591A;S602D]/2Q*^ but not WT mice develop kyphosis, shown at 36 weeks of age. (B) Monthly weights, n = 13–19. (C) Barnes maze at 14 weeks of age. (D) Minute ventilation as measured via plethysmography at 40 weeks of age. (E) Survival analysis. (F) Up and downregulated differentially expressed genes (DEGs) by brain region. Dysregulated genes were determined as having an adjusted p value < 0.05. Rescued genes were determined as having an adjusted p value < 0.05 in the *Atxn1*^*154Q/2Q*^ mouse model and an adjusted p value > 0.05 in the *Atxn1*^*154Q[V591A;S602D]/2Q*^ mouse model. Bulk RNA sequencing was conducted at 10 weeks of age, n = 3–4. (G) UpSet plot of dysregulated genes in *Atxn1*^154Q/2Q^ mouse model by brain region. (H) Dot plot of KEGG pathways enriched in *Atxn1*^*154Q/2Q*^ DEGs by brain region. (I) Dot plot of KEGG pathways enriched in *Atxn1*^*154Q[V591A;S602D]/2Q*^ DEGs by brain region. (J) Jitter plot of CIC motif enrichment in *Atxn1*^*154Q/2Q*^ and *Atxn1*^*154Q[V591A;S602D]/2Q*^ DEGs by brain region. The black dot indicates % DEGs with CIC motif within 1 kb of the transcriptional start site from each respective RNA-seq dataset. Colored jitter plot represents the % of non-DEGs with motif calculated over 10,000 random iterations. For each assay, a minimum of 8 mice were used unless otherwise specified. Two-way ANOVA with Tukey’s multiple comparisons was used for (B); one-way ANOVAs with Tukey’s multiple comparisons test were used for (C) and (D); Mantel-Cox log-rank was used for (E). In each case, *, **, ***, ****, and ns denote p < 0.05, p < 0.01, p < 0.001, p < 0.0001, and p > 0.05, respectively. All data are represented as mean ± SEM. See also Figure S1.

After determining the role of the ATXN1-CIC complex in the cerebellum, we tested if ablation of this complex improves SCA1 phenotypes brain-wide. We first characterized two prominent and robust general SCA1 phenotypes, the formation of the hunched posture (kyphosis) and the failure to gain weight after 8 weeks of age,^10^ both of which persisted in *Atxn1*^*154Q[V591A;S602D]/2Q*^ mice (Figures 2A and 2B).

Next, we evaluated learning and memory deficits, which are observed in some patients with SCA1, particularly in cases with juvenile onset.^12^ We performed the Barnes maze, a hippocampus-dependent memory task, where mice use spatial cues to find an escape tunnel.^13^ We found that *Atxn1*^*154Q/2Q*^ and *Atxn1*^*154Q[V591A;S602D]/2Q*^ mice both performed poorly relative to WT mice, needing more time to locate the tunnel (Figure 2C). SCA1 patients display respiratory dysfunction, which is recapitulated in the SCA1 knockin mouse model.^14^ We performed plethysmography to measure breathing and found reduced minute ventilation in *Atxn1*^*154Q/2Q*^ mice, which was improved to WT levels in the *Atxn1*^*154Q[V591A;S602D]/2Q*^ genotype (Figures 2D and S1H-I). SCA1 is typically lethal due to bulbar dysfunction 10–30 years after symptom onset.^3^ Therefore, we next assessed the lifespan and found that *Atxn1*^*154Q[V591A;S602D]/2Q*^ mice lived 9 weeks longer than *Atxn1*^*154Q/2Q*^ mice (Figure 2E).

To understand the contributions of the ATXN1-CIC complex to SCA1 transcriptional changes brain-wide, we conducted RNA-seq in five brain regions (cerebellum, hippocampus, brainstem, cortex, and striatum) at 10 weeks of age. We found differentially expressed genes (DEGs) in all brain regions evaluated in the *Atxn1*^*154Q/2Q*^ mice compared with WT and rescue ranging from 53% to 88% in the *Atxn1*^*154Q[V591A;S602D]/2Q*^ mice (Figure 2F). The brainstem demonstrated the fewest DEGs, likely because degeneration in this region occurs later than in other regions, and therefore, brainstem data could not be used for all transcriptional analyses. Most DEGs were brain-region specific (Figure 2G); however, KEGG analysis revealed that there is some overlap in the pathways altered in SCA1 (Figure 2H). CIC regulates genes in the MAPK pathway,^15^ and we see enrichment of this pathway in four brain regions in *Atxn1*^*154Q/2Q*^ mice (Figure 2H); however, this pathway is only rescued in the cerebellum in *Atxn1*^*154Q[V591A;S602D]/2Q*^ mice (Figure 2I), suggesting a unique role of CIC in the cerebellum. We hypothesized that the partial transcriptional rescue is due to improvements in genes that are regulated by CIC. To test this, we quantified enrichment of the CIC consensus motif within the *Atxn1*^*154Q/2Q*^ and *Atxn1*^*154Q[V591A;S602D]/2Q*^ DEGs. We found that the CIC motif is enriched in *Atxn1*^*154Q/2Q*^ DEGs in three of the four brain regions assessed, the cerebellum, hippocampus, and striatum (Figure 2J). This enrichment is lost in the *Atxn1*^*154Q[V591A;S602D]/2Q*^ DEGs, suggesting that many CIC dysregulated genes in these regions are corrected (Figure 2J).

These data demonstrate that broad disruption of the ATXN1-CIC complex partially improves SCA1 neurological phenotypes. The incomplete rescue indicates the ATXN1-CIC complex cannot be the sole driver of SCA1 pathogenesis brain-wide and that other factors are involved in disease. Hence, we rationalized that molecular analysis of the *Atxn1*^*154Q[V591A;S602D]/2Q*^ model could give insight into CIC-dependent and independent transcriptional changes in SCA1 to uncover additional drivers of disease.

### Molecular characterization of *Atxn1*^*154Q[V591A;S602D]/2Q*^ mice demonstrates CIC-dependent and independent contributions to SCA1 pathogenesis

The ATXN1-CIC complex is thought to cause toxicity in SCA1 through a gain-of-function mechanism that results in hyper-repression of CIC target genes.^6^ Surprisingly, the genome-wide CIC binding pattern has never been profiled in SCA1. To pinpoint the CIC targetome in SCA1, we conducted cleavage under targets and release using nuclease (CUT&RUN) in the mouse cerebellum^16,17^ using a CIC polyclonal antibody.^7,18^ We utilized four genotypes for this experiment: WT, *Atxn1*^*154Q/2Q*^*, Atxn1*^*154Q[V591A;S602D]/2Q*^, and *En1-Cre;Cic*^*fl/fl*^. The *En1-Cre; Cic*^*fl/fl*^ genotype ablates *Cic* expression throughout the cerebellum, serving as a negative control in our experiment.^19^ We identified peaks of CIC binding by annotating regions where WT and *Atxn1*^*154Q/2Q*^ had significantly higher signal compared with *En1-Cre;Cic*^*fl/fl*^ (Figure S2A).

**Figure 3.**
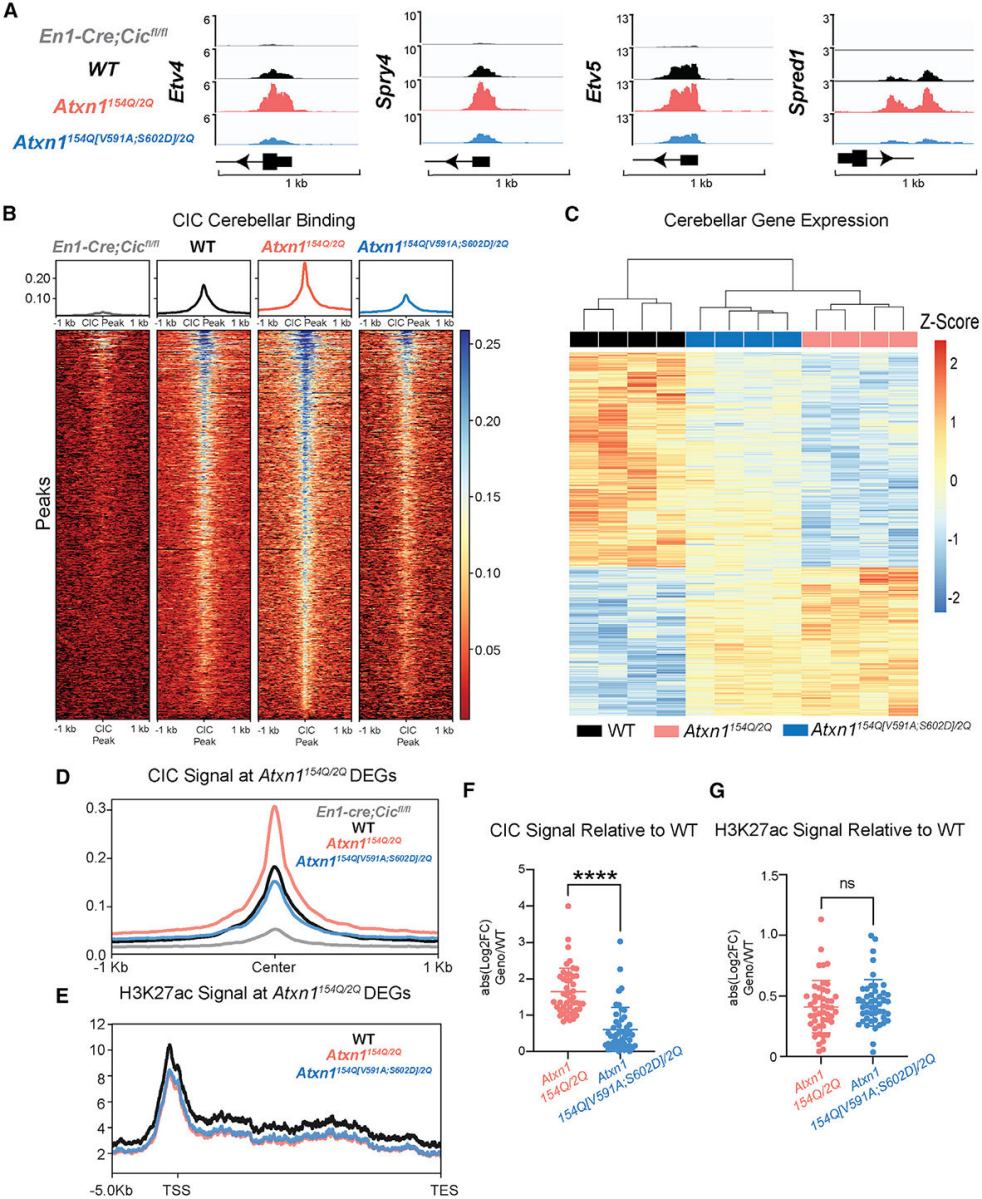
Molecular characterization of *Atxn1*^*154Q[V591A;S602D]/2Q*^ mice demonstrates CIC-dependent and independent contributions to SCA1 (A) Integrative Genomics Viewer (IGV) tracks displaying CIC binding of validated CIC targets *Etv4, Spry4, Etv5,* and *Spred1* from CUT&RUN, 10 weeks of age, n = 3–4. (B) Heatmap of CIC signal at CIC peaks. (C) Heatmap of cerebellar *Atxn1*^*154Q/2Q*^ differentially expressed genes (DEGs). (D) CIC signal plot at *Atxn1*^*154Q/2Q*^ DEGs with CIC peaks. (E) H3K27ac signal plot throughout promoter and gene body at *Atxn1*^*154Q/2Q*^ DEGs. (F) Quantification of CIC signal using the absolute value log_2_ fold change of *Atxn1*^*154Q/2Q*^ and *Atxn1*^*154Q[V591A;S602D]/2Q*^ compared with WT. (G) Quantification of H3K27ac signal using the absolute value log_2_-fold change of *Atxn1*^*154Q/2Q*^ and *Atxn1*^*154Q[V591A;S602D]/2Q*^ compared with WT. Mann-Whitney tests were used for (F) and (G). In each case, *, **, ***, ****, and ns denote p < 0.05, p < 0.01, p < 0.001, p < 0.0001, and p > 0.05, respectively. See also Figure S2.

To validate our results, we first assessed CIC binding at established CIC targets.^15^ We identified *Etv4, Spry4, Etv5,* and *Spred1* in the top 10 most significantly identified peaks, and as expected, these are upregulated in *En1-Cre;Cic*^*fl/fl*^ RNA-seq data (Figures 3A and S2B). Next, we assessed genome-wide CIC binding in the 2,079 peaks identified in the *Atxn1*^*154Q/2Q*^ mice, and 64% of these peaks fell in a promoter region within 1 kb of a gene transcriptional start site (Figure S2C). We generated a *de novo* CIC consensus motif that revealed an enriched 14-mer motif which was significantly similar to the previously published CIC consensus motif (Figure S2D).^15,20^ We detected little to no CIC binding in the *En1-Cre;Cic*^*fl/fl*^, demonstrating the specificity of the identified peaks (Figure 3B). The highest CIC binding was identified in the *Atxn1*^*154Q/2Q*^ genotype, consistent with a gain of function of the complex. Importantly, CIC binding is normalized to WT levels in *Atxn1*^*154Q[V591A;S602D]/2Q*^ (Figure 3B), demonstrating that the interaction of polyQ-expanded ATXN1 with CIC is critical for CIC aberrant DNA binding in SCA1. Moreover, *Atxn1*^*154Q[V591A;S602D]/2Q*^ CIC binding is slightly less than WT, likely due to only one ATXN1 allele able to bind to CIC versus two alleles in WT.

Next, we evaluated CIC peaks in relation to transcriptional changes. Cerebellar RNA-seq showed 1,647 DEGs in the *Atxn1*^*154Q/2Q*^ mice (Figures 2F and 3C). We intersected these with CIC peaks and found that 131 altered *Atxn1*^*154Q/2Q*^ DEGs contained a CIC peak. When we split DEGs by their directionality, we find an enrichment of CIC peaks in the downregulated genes but not the upregulated genes, highlighting the role of CIC as a transcriptional repressor (Figure S2E). Similar to the genome-wide pattern, CIC binding is enhanced at differentially expressed loci in the *Atxn1*^*154Q/2Q*^ mice and reduced to WT levels in the *Atxn1*^*154Q[V591A;S602D]/2Q*^ mice (Figures 3D and 3F). CIC functions as a transcriptional repressor via recruitment and interaction with histone deacetylase complexes (HDACs) that remove histone acetyl marks leading to reduced transcriptional activity.^15^ Therefore, we measured levels of H3K27ac, a marker inversely correlated with CIC chromatin binding.^15^ We observed lower H3K27ac occupancy at SCA1 DEGs in the *Atxn1*^*154Q/2Q*^ genotype compared with WT; however, surprisingly, the *Atxn1*^*154Q[V591A;S602D]/2Q*^ genotype only exhibited a modest increase in H3K27ac relative to *Atxn1*^*154Q/2Q*^ (Figure 3E). At SCA1 DEGs with a CIC peak, we see no change in acetylation between *Atxn1*^*154Q/2Q*^ and *Atxn1*^*154Q[V591A;S602D]/2Q*^ (Figure 3G). The sustained reduction in acetylation may account for the incomplete transcriptional rescue despite restoration of CIC binding to WT levels in the *Atxn1*^*154Q[V591A;S602D]/2Q*^ model. These data led us to consider other factors that might interact with ATXN1 to regulate the transcriptome in SCA1.

### Additional ATXN1 nuclear interactors may regulate SCA1 transcriptional targets

**Figure 4.**
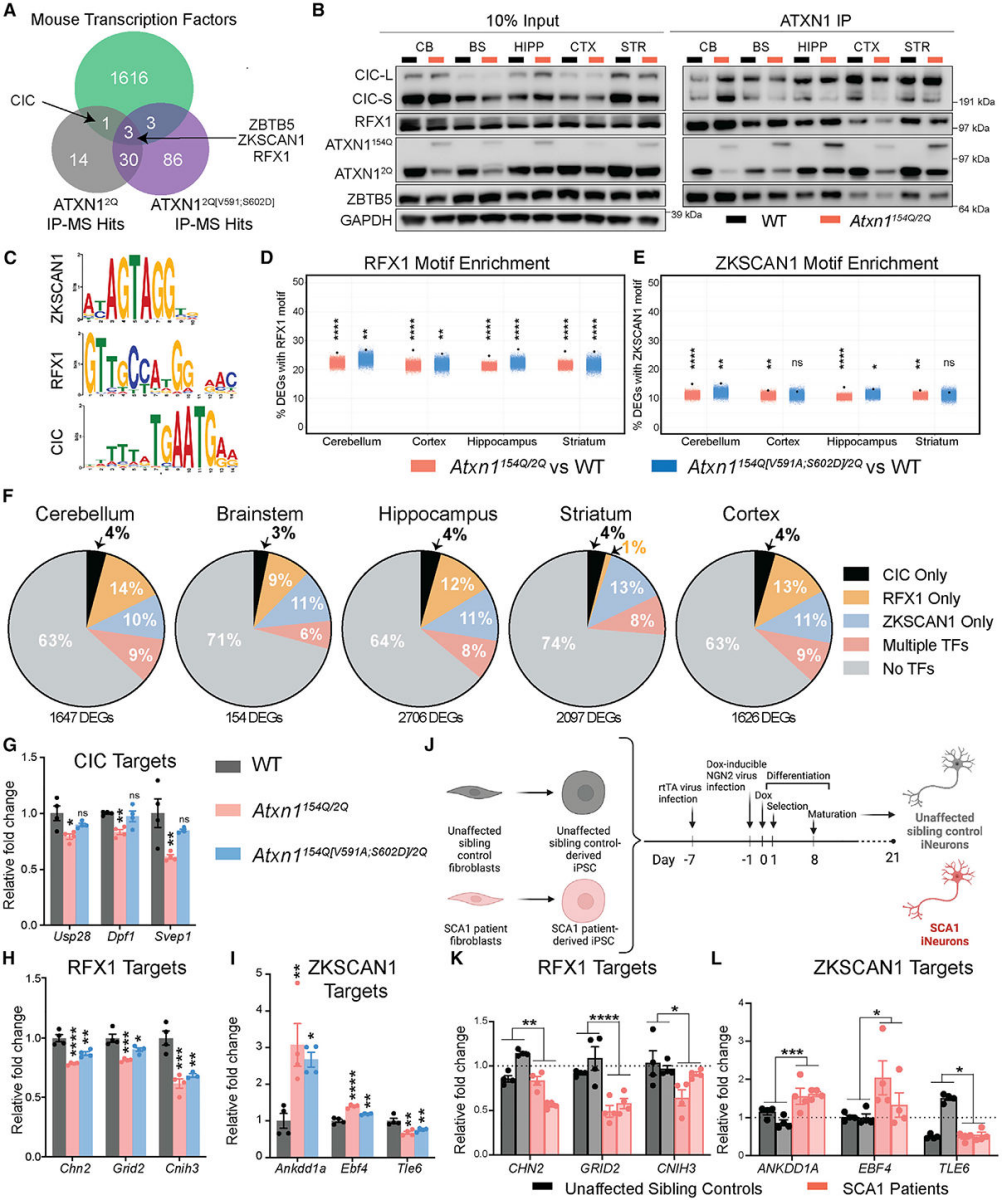
Additional ATXN1 interacting transcription factors regulate genes altered in SCA1 (A) Proteins identified by IP-MS of ATXN1 in cerebellar tissue at 4 weeks of age and intersected with mouse transcription factors. (B) IP of ATXN1 and blotting for transcription factors CIC, RFX1, and ZBTB5 in 4-week cerebellar tissue. (C–E) (C) Comparison of available consensus motifs for RFX1, ZKSCAN1, and CIC. Jitter plot of (D) RFX1 and (E) ZKSCAN1 motif enrichment in *Atxn1*^*154Q/2Q*^ and *Atxn1*^*154Q[V591A;S602D]/2Q*^ DEGs by brain region. The black dot indicates % DEGs with motif within 1 kb of the transcriptional start site from each respective RNA-seq dataset. Colored jitter plot represents the % of non-DEGs with motif calculated over 10,000 random iterations. (F–I) (F) Pie charts of *Atxn1*^*154Q/2Q*^ DEGs parsed by which contain CIC, RFX1, or ZKSCAN1 peaks analyzed across brain regions. “Multiple TFs” refers to *Atxn1*^*154Q/2Q*^ DEGs that contain transcription factors motifs from two or more factors. Bar plots of relative normalized RNA-seq gene counts of *Atxn1*^*154Q/2Q*^ DEGs in the cerebellum that contain a (G) CIC, (H) RFX1, or (I) ZKSCAN1 peak. (J–L) (J) Cartoon demonstrating differentiation process of SCA1 and healthy patient control iPSCs into iNeurons. Cartoon generated using Biorender.com. Bar chart demonstrating RT-qPCR RNA expression data from genes regulated by (K) RFX1 or (L) ZKSCAN1 in human iPSC-derived iNeurons. Each bar represents an original biological sample collected (2 healthy controls and 2 SCA1 patients), and each data point within the bar represents a line generated from that sample (n = 4). One-way ANOVAs with Dunnett’s multiple comparisons test were used for (G), (H), and (I). t tests were used in (K) and (L). in each case, *, **, ***, **** and ns denote p < 0.05, p < 0.01, p < 0.001, p < 0.0001 and p > 0.05, respectively. All data are represented as mean ± SEM. See also Figure S3.

The inability of the *Atxn1*^*154Q[V591A;S602D]*^ allele to fully rescue transcriptional or epigenetic changes in SCA1, coupled with the fact that polyQ-expanded ATXN1 nuclear localization is critical for its toxicity,^21^ led us to hypothesize that there must be additional nuclear proteins contributing to SCA1 pathogenesis. We rationalized that it is best to identify non-CIC interactors in a context where ATXN1 is free from its interaction with CIC, given how abundant these two proteins are and how strong the interaction is between them. To this end, we bred the *Atxn1*^*2Q[V591A;S602D]/2Q*^ mice to homozygosity and used these mice for immunoprecipitation followed by mass spectrometry (IP-MS) to identify ATXN1 interactors in an unbiased manner in the absence of ATXN1-CIC binding (Figure S3A). We searched the MS data for transcription factors, given the alteration in H3K27ac signal and the incomplete transcriptional rescue in the *Atxn1*^*154Q[V591A;S602D]/2Q*^ model. We found CIC bound to ATXN1^2Q^ but not ATXN1^2Q[V591;S602D]^, further validating the functionality of the V591A;S602D mutations (Figure 4A). We focused on the three transcription factors that interact with both ATXN1^2Q^ and ATXN1^2Q[V591A;S602D]^ and are expressed brain-wide in human and mouse: zinc finger with KRAB and SCAN domains 1 (ZKSCAN1), zinc finger and BTB domain containing 5 (ZBTB5), and regulatory factor X 1 (RFX1) (Figures 4A, S3-C). We found commercial grade antibodies for ZBTB5 and RFX1 and validated these interactions in the presence of the polyQ-expanded allele (Figure 4B). Further, ZBTB5 and RFX1 are transcriptional repressors, which, such as CIC, have been shown to interact with HDAC complexes.^22,23^

The consensus binding motifs were known for two of these transcription factors, RFX1 and ZKSCAN1, and are distinctly different from the CIC motif (Figure 4C). We next tested if the RFX1 or ZKSCAN1 motifs are enriched in *Atxn1*^*154Q/2Q*^ or *Atxn1*^*154Q[V591A;S602D]/2Q*^ DEGs. RFX1 and ZKSCAN1 are enriched in all brain regions in the *Atxn1*^*154Q/2Q*^ genotype, and this enrichment is sustained in most brain regions in the *Atxn1*^*154Q[V591A;S602D]/2Q*^ genotype (Figures 4D and 4E). In comparison, the CIC binding motif was enriched brain-wide in the *Atxn1*^*154Q/2Q*^ genotype, but this disappeared in the *Atxn1*^*154Q[V591A;S602D]/2Q*^ mouse (Figure 2J). Therefore, although many CIC-regulated DEGs are rescued in the *Atxn1*^*154Q[V591A;S602D]/2Q*^ mouse, there are still many genes whose expression is likely controlled by either RFX1 or ZKSCAN1, or other transcription factors, that remain dysregulated. Next, we analyzed published ChIP-seq data for RFX1 and ZKSCAN1 to determine genes with peaks for each factor.^24,25^ We then tested which *Atxn1*^*154Q/2Q*^ DEGs contained CIC, RFX1, or ZKSCAN1 peaks, either uniquely or in combination, and found that CIC alone is predicted to only regulate only 3%–4% of DEGs, highlighting the potential important contributions of these new transcription factors to SCA1 (Figure 4F). Investigation of the top cerebellar DEGs regulated by each factor in our RNA-seq data revealed that genes regulated by CIC are rescued in the *Atxn1*^*154Q[V591A;S602D]/2Q*^ mouse (Figure 4G), whereas genes regulated by only RFX1 or ZKSCAN1 are not rescued in the *Atxn1*^*154Q[V591A;S602D]/2Q*^ mouse (Figures 4H-I), as these factors can still bind polyQ-expanded ATXN1. Last, to determine if RFX1 and ZKSCAN1 target genes are altered in a human SCA1 model, we tested the expression of ChIP-validated target genes in human iNeurons of healthy controls and SCA1 patients (Figures 4J and S3D). Indeed, genes altered in a SCA1 mouse model with RFX1 and ZKSCAN1 peaks are also altered in human iNeurons (Figures 4K-L).

These data demonstrate that ATXN1 interacts with a host of transcription factors and that these additional factors might co-regulate genes dysregulated in SCA1 and contribute to pathogenesis in mouse and human models.

## DISCUSSION

A key question in the field of neurodegeneration is what causes selective neuronal vulnerability in face of mutations in genes that encode widely expressed proteins. In this study, we sought to address this question in a monogenic disorder using a mouse model that reproduces the spatial and temporal expression of *Atxn1* seen in human disease. By mutating polyQ-expanded *Atxn1* in the knockin SCA1 mouse to prevent its interaction with its partner CIC throughout the brain, we had the tools to determine if the pathogenic mechanism driving toxicity in cerebellar PCs is identical across all affected brain regions. We discovered that ablation of the ATXN1-CIC complex partially improves some neurological phenotypes, including motor coordination, respiration, and lifespan but does not improve learning and memory, kyphosis, or body weight. We detected full rescue of the structure and function of the cerebellar PC microcircuit, indicating that the pathogenic mechanism in SCA1 varies by cell type. These data demonstrate the ATXN1-CIC complex is critical for cerebellar PCs, and although it contributes to polyQ-expanded toxicity in other brain regions, it is not the sole driver of disease. These results also suggest that either cerebellar cell types other than PCs, or other brain regions, contribute to the motor incoordination phenotype seen on the rotarod assay. One possibility may be the striatum, as this brain region is involved in motor planning and coordination, and striatal putamen degeneration has recently been identified in SCA1 patients.^26^

Because CIC is a transcriptional repressor that binds DNA, it afforded us the opportunity to dissect the molecular contributions of CIC to SCA1. We profiled the genome-wide binding pattern of CIC in cerebella of healthy and SCA1 mice and intersected these data with transcriptomic data from the same tissue. We discovered that CIC binding is increased in the *Atxn1*^*154Q/2Q*^ mouse, with the H3K27ac signal decreased concomitantly, supporting the role of CIC as a transcriptional repressor in SCA1.^6^ Most intriguing was the discovery that CIC binding is normalized in the *Atxn1*^*154Q[V591A;S602D]/2Q*^ mouse; however, many genes remained altered at the RNA level, and these genes sustained a reduction in the H3K27ac mark. This led us to propose that other transcriptional regulators must be involved in SCA1 pathogenesis. Using unbiased proteomics, we identified three novel ATXN1 interacting transcription factors: RFX1, ZBTB5, and ZKSCAN1, which together with CIC, are predicted to regulate ~33% of the altered genes in SCA1 brain-wide. Further, ChIP-validated target genes of these factors are altered in both mouse and human SCA1 models of disease.

There are several lessons that we learned from the studies reported here. First, CIC seems to be the main driver of pathogenesis in one cell type, cerebellar PCs, but is a less critical contributor to other cell types. Second, ATXN1 has many interactions that are likely contributing to pathogenesis, even within one brain region. Third, combining epigenetic profiling with gene expression helped narrow our focus to transcription factors. Last, the most important lesson we learned from these studies is the complexity of molecular mechanisms driving regional and cellular vulnerability. Here, the transcriptional changes and interactions have been interrogated deeply in one tissue, the cerebellum. As we contemplate pathogenic mechanisms in the hippocampus, brainstem, and striatum, additional regional interactor studies will be needed, although some of the mechanisms identified in the cerebellum might apply to these regions.

Our findings within SCA1 are likely not unique and open the possibility of such mechanistic complexity for other polyQ diseases and the broader class of neurodegenerative diseases such as Alzheimer’s and Parkinson’s disease. The data also highlight the importance of having disease models that express the mutant protein in the correct spatial and temporal pattern. The studies in this paper make it abundantly clear that what can be learned from transgenic mice is limited to the cell type studied and cannot be generalized to other cell types or tissues. Although it might seem daunting, the discovery of diverse mechanisms of disease opens new opportunities to intervene and modulate the course of these disorders.

## STAR★METHODS

### RESOURCE AVAILABILITY

#### Lead contact

Further information and requests for resources and reagents should be directed to and will be fulfilled by the lead contact, Dr. Huda Y. Zoghbi (hzoghbi@bcm.edu).

#### Materials availability

The mouse lines generated in this study have been deposited to Jackson Labs. *Atxn1*^*2Q[V591A;S602D]/2Q*^ (Stock # 037674) and *Atxn1*^*154Q[V591A;S602D]/2Q*^ (Stock # 037673).

#### Data and code availability

All DNA and RNA sequencing FASTQ data files from the RNA-sequencing and CUT&RUN experiments have been deposited to GEO (GEO # GSE218302). This paper analyzes existing, publicly available data *(Engrailed-1-Cre;Cic*^*flox/flox*^ RNA-seq GEO # GSE108254, RFX1 ChIP-seq GEO # GSM594584, and ZKSCAN1 ChIP-seq GEO # GSM1003779). This paper does not report any original code. Any additional information required to reanalyze the data reported in this paper is available from the lead contact upon request.

### EXPERIMENTAL MODEL DETAILS

#### Mouse husbandry

All mice were backcrossed to C57BL/6J background. Mice were housed and maintained in the animal facilities at Baylor College of Medicine. The mice were kept on a 12 hr light/12 hr dark cycle. Animal care and experimental procedures were approved by the institutional animal care and use committee of Baylor College of Medicine, according to the US National Institutes of Health Guidelines.

### METHOD DETAILS

#### Generation of *Atxn1*^*154Q[V591A;S602D]/2Q*^ and *Atxn1*^*2Q[V591A;S602D]/2Q*^ mouse models

*Atxn1*^*2Q[V591A;S602D]/2Q*^ and *Atxn1*^*154Q[V591A;S602D]/2Q*^ mice were generated via CRISPR/Cas9-mediated gene editing.^40,41^ Please note that human V591 and S602 correspond with mouse V620 and S631, respectively. The human nomenclature was maintained for consistency between transgenic mice and knock-in animals, and to avoid discrepancies with alterations in CAG repeat length.^9^ The crRNA (5′-GGTGGAGGACCTGAAGACGG-3′) was selected based on the highest quality score and a low number of off-target sites using the crispr.mit.edu homepage, and the same crRNA was used for both models. The crRNA was purchased from Integrated DNA Technologies (IDT) from the Alt-R CRISPR-Cas9 genome editing section. A single-stranded oligonucleotide (ssODN) was purchased from IDT as a 4 nmol Ultramer with desalting for homology directed repair to introduce the V591A and S602D amino acid changes, with additional synonymous mutations in *Atxn1* (5′-CAAACTGTATCACGGCCACCCCGGGGCTGTGGCTCTCCTCGATTCT CTCCACAGTACTGGAGTCGATCTTGAGGTCATTGCTAATCTCTGCAtcCTGGATGAAATCtTCtGTtTTtAGaTCtTCggCCTTCTTCAGCTCCCCGTTGGCCAGCTGGATGATGGA-3′; modified nucleotides are written in lowercase). The additional synonymous mutations were used to alter the PAM motif to prevent further editing as well as to allow for genotyping by differential primer hybridization. Stock Alt-R^®^ CRISPR-Cas9 tracrRNA 5 nmol (catalog # 1072532) and Alt-R^®^ S.p. HiFi Cas9 Nuclease V3 (catalog # 1081060) were purchased from IDT. The tracrRNA was diluted in T_10_E_0.1_ buffer to 20 μM and the Cas9 was diluted in Cas9 dilutant buffer (300 mM NaCl, 10 mM Tris HCl, 0.1 mM EDTA, 1 mM DTT, 500 μg/ml BSA, 50% glycerol, pH 7.4). All reagents were stored in −80°C.

4-5 week-old female C57BL/6J WT mice were purchased from Jackson Lab and super-ovulated via injection of 5 IU of PMSG (Thermo Fisher) and 47 hrs later with 5 IU HCG (Thermo Fisher) in 0.9% NaCl. Upon overnight breeding with *Atxn1*^*154Q/2Q*^ males, the females’ ova were dissected.

Prior to the day of injection, the oligo mixture was prepped by combining 3 μl of our designed crRNA (20 μM) with 3 μl of tracrRNA (20 μM) and incubating for 10 minutes at RT. Next, 1.225 μl of 1 μg/ul ssODN was added, and the final volume was brought up to 95 μl in T_10_E_0.1_ buffer. This oligo mixture was kept at −80°C until the day of injection. On the day of injection, the oligo mixture was thawed, and 5 μl of Cas9 was added. The tube was lightly mixed, put on wet ice, and taken to the Baylor College of Medicine (BCM) Genetically Engineered Mouse Core. 2 pL was injected into the pronucleus of extracted ova. The ova were then transferred into oviducts of pseudopregnant ICR (CD1) females by the BCM GERM Core.

Upon birth, the following primers were used to distinguish between the unmodified (WT) and modified (V591A;S602D) alleles: WT_V591A;S602D_CRISPR_For (5′-AGCCACGGCCTTCTACGCTG3′), WT_CRISPR_Rev (5′-ACTCTGGATGAAATCCTCCGTCTTCAGGTCCTCCACCTTCTTCAGC-3′) and V591A;S602D_CRISPR_Rev (5′-cCTGGATGAAATCtTCtGTtTTtAGaTCtTCggCCTTCTTCAGC-3′). Note that the forward primer is the same for both reactions. Positive founders were backcrossed to C57BL/6J WT animals (Jackson Laboratory) for a minimum of five generations, and the correct sequence of the F1 offspring was confirmed using Sanger sequencing (Genewiz). Further, offspring from two independent CRISPR modified founder lines were tested on the rotarod assay and demonstrated similar results. Segregation of the V591A;S602D mutations with the *Atxn1*^*154Q*^ or *Atxn1*^*2Q*^ allele and further confirmation via immunoprecipitation confirmed which *Atxn1* allele the mutations were on.

#### RNA extraction and quantitative real time PCR

RNA from cerebella was isolated from 4-week-old mice (n=3 per genotype). Mice were crossed to *Atxn1*^*2Q/−*^ animals in order to quantify RNA levels of a single *Atxn1* allele for Figure 1C.^28^ Total RNA was isolated using the miRNeasy Mini Kit from Qiagen following the manufacturer’s instructions. RNA was quantified using NanoDrop 1000 (Thermo Fisher), and random-primed cDNA was prepared from 1 μg of total RNA using M-MLV Reverse Transcriptase (Invitrogen). qRT-PCR was then performed with PowerUp SYBR Green Master Mix (Applied Biosystems), and samples were run on a real-time PCR detection system (BioRad CFX96). All samples were analyzed in triplicate, and *Atxn1* and Cic expression levels were normalized to the expression of the housekeeping gene *Gapdh*. Primer sequences are included in the Table S1.

#### Protein extraction and western blot of brain tissues

Homogenates of different brain regions from 4-week-old mice were prepared by Dounce homogenization (50x with 2mL size homogenizer, using Pestle B) in 1 mL NETN buffer (100 mM NaCl, 20 mM Tris-HCl, pH 8.0, 0.5mM EDTA, 1.5% NP-40, 1X protease inhibitor (Roche), 1X phosphatase inhibitor (Sigma)). Samples were sonicated for a total of 15 cycles, incubated for 30 min at 4°C, and spun at 13,000 rpm at 4°C for 15 min. Protein concentrations of the supernatant were measured using the Pierce BCA Protein Assay Kit (Thermo Fisher Scientific). Samples were diluted and prepared in NuPAGE sample reducing agent (Invitrogen) and NuPAGE LDS Sample Buffer (Invitrogen). The samples were boiled for 10 min and then run on NuPAGE 4-12% Bis-Tris 1.5mm 15-well gels (Thermo Fisher, NP0336BOX). The proteins were subsequently transferred to 0.2 μm nitrocellulose membranes (Amersham #10600004) for 2 hr at 0.32 Amps. After blocking for 1hr at room temperature with 5% milk in 1xTBST, membranes were washed 3x for 10min at RT in 1xTBST, then probed overnight at 4°C with anti-ATXN1 (in house, rabbit, 11750VII, 1:2,000),^27^ anti-CIC (rabbit in-house, 1:1,000), anti-RFX1 (rabbit Bethyl Cat# A303-043A, 1:2,000), anit-ZBTB5 (rabbit Atlas Antibodies Cat# HPA021521, 0.4 μg/ml), and anti-GAPDH (mouse, Millipore; 6C5, 1:10,000) in 1:1 Odyssey Blocking Buffer TBS (Licor) in tris buffered saline (5 mM Tris pH 7.5, 120 mM NaCl) with 0.1% Tween-20 (TBST). The secondary antibody used to detect anti-ATXN1 and anti-CIC was Goat Anti-Rabbit-HRP conjugate (Bio-Rad #170-5046, 1:10,000), and the secondary used to detect anti-GAPDH was Donkey Anti-Mouse IgG (Jackson ImmunoResearch #715-035-150, 1:10,000), all in 5% milk in 1xTBST. The membranes were washed three times with TBST and then imaged using the GE Amersham Imager 680.

#### CIC immunoprecipitation

Brain regions from 4-week-old mice were lysed in 1mL NEMT Buffer (50 mM Tris pH 7.5, 0.5% Np-40, 150 mM NaCl, 1mM EDTA) supplemented with fresh 1X protease inhibitor (Roche) and 1X phosphatase inhibitor (Sigma) using a Dounce homogenizer (50x with 2mL size homogenizer, using Pestle B). Lysate was incubated on ice for 20 mins and then centrifuged at max speed for 20 mins at 4°C. 15μl of Protein G Dynabeads (Invitrogen, 10004D) were washed 3x with 500μl of 1xPBS and then incubated at RT for 40 mins with 600μl 5% BSA in 1x PBS with either 1μl of normal rabbit IgG (EMD Millipore #12-370) or 5 ml of rabbit anti-CIC (in house).^7,18^ Beads were washed 2x with 500μl NEMT on ice, and then 300μl of the lysate was loaded on the washed beads and incubated at 4°C for 45 mins. Beads were washed 4x with 500μl NEMT on ice and in second wash, beads were moved to a new tube. After final wash, loading buffer was added and beads were boiled for 10 mins at 95°C. After elution, beads were removed and samples were ran on NuPAGE 4-12% Bis-Tris 1.5mm 15-well gels (Thermo Fisher, NP0336BOX).

#### Behavioral tests

All behavioral analyses were performed during the light phase of the 12 hr light/dark cycle by an experimenter blinded to the genotype of the mice. The mice had access to food and water *ad libitum* except during tests. All mice were age-matched within experiments and littermate controls were used when possible. For each test, the mice were habituated for 30 min in the test room before testing. Testing was done at a room brightness of 200 lux with white noise playing at 60dB.

##### Rotarod

The rotarod test was performed at seven, fifteen and twenty-four weeks of age to evaluate coordination and motor skill acquisition (Type 7650; Ugo Basile). The mice were placed on the rotating rod (3 cm diameter, 30 cm long) for 4 trials every day for a period of 4 days. Each trial lasted a maximum of 10 min. The rod accelerated from 4 to 40 rpm in 5 min, and remained at 40 rpm for the remaining 5 min. The time the mice took to fall was recorded. Two subsequent rotations around the rod were also counted as a fall.

##### Barnes maze

Barnes Maze was done as described^13^ with the following adaptation. Mice were allowed to habituate in the room minimum of 30 mins prior to testing. During training, mice were placed on Barnes Maze twice a day, for 3 min trials. Mice that did not find the escape tunnel at the end of the training trial were placed into the tunnel. Training was done for 4 consecutive days, with and inter-trial interval of at least 30 mins. On the fifth day, the escape hole was covered, and mice were placed on the Barnes maze for 3 mins. Probe latency time (amount of time it took mice to first pass the probe area) and speed were recorded. Speed was confirmed to not be significantly different to ensure that speed does not confound the latency time.

##### Plethysmography

Whole-body plethysmography (Buxco) was performed as described previously.^14,42^ Mice at 40weeks of age were habituated to the chambers for 1 hr before recording the respiration for 30 min. Air was pumped through the chambers at a rate of 0.5 L/min. Breath waveforms were identified using Phonemah 3 software (DSI), and the breathing rate and tidal volume were subsequentially analyzed using a customized MATLAB (MathWorks) code. As mouse movements can induce artifacts, breaths with an inspiratory time under 0.025 sec, expiratory time over 10 sec, or calculated expiratory tidal volume more than twice the inspiratory tidal volume were excluded. A sliding window of 200 breaths was used to filter out intervals during which more than 10% of breaths were taken at a rate faster than 600 breaths/min. Inter-breath interval irregularity (IBII) was defined as IBII=abs[breath length(*n*+1)–breath length(*n*)]/breath length(*n*) (abs: the absolute value; *n*: chronological number of a recorded breath). Mice with less than 100 reliable recorded breaths were excluded from the analysis.

#### Histology

Deeply anesthetized mice were perfused with PBS followed by 4% PFA in 1xPBS. Isolated brain tissues were post-fixed with 4% PFA in PBS for 48 h at 4°C, followed by 30% sucrose in 1xPBS for cryoprotection at 4°C. The brain tissues were then embedded in Tissuetek OCT compounds (Sakura, #4583) and sagittally sectioned with 30 μm thickness starting at 1.725 mm and freely floated in PBS for staining. The floating cryosections were washed 3x with 1xPBS and then blocked with 10% NGS in 0.2% Triton X-100 for an hour at RT. Mouse anti-Calbindin antibody (Swant, McAB300, 1:600) was added into the blocking buffer and incubated for 24 h at 4°C with gentle shaking. After 3x of washing with 1x PBS, the sections were then incubated with anti-mouse secondary antibody conjugated with Alexa Fluor 488 (Invitrogen, A11029, 1:500) for 24 hr at 4°C with gentle shaking. The stained sections were washed 3x with 1xPBS, counterstained with Dapi for 5 min (1:5000, ThermoFisher D1306) and mounted on a slide with Vectashield antifading mounting media (Vector Laboratories, H-1000). Fluorescence imaging of the tissues was performed on a Nikon Eclipse Ti2-E confocal microscope. The whole cerebellum image of each stained section was obtained using a tile-scanning function, and the thickness of the two molecular layers between the lobule V and VI were measured using an ImageJ software (Fiji, version 2.1.0/1.53c). The procedure for performing free-floating immunohistochemistry with CAR8 and IP3R1 has been described previously.^43,44^ Blinded experimenters performed the immunohistochemistry, imaging, and quantification of puncta in the cerebellar nuclei. Purkinje cell bodies, processes, and terminals were immunolabeled with both 1:500 rabbit anti-carbonic anhydrase VIII (CAR8) (12391-1-AP; Proteintech) and 1:500 rabbit anti-IP3R1 (PA1-901; Invitrogen) primary antibodies followed by 1:1500 Alexa-350 secondary (A10039; Invitrogen). Photomicrographs of stained tissue were collected using a Zeiss Axio Imager.M2 microscope equipped with a Zeiss AxioCam MRm camera and apotome. Single-plane 40x images of fastigial nuclei were acquired for both CAR8/IP3R1 and VGAT quantification. One image per stain and area of interest was analyzed per animal. Images were prepared for analysis using FIJI software (ImageJ).^45-47^ Our method for puncta counting using FIJI software has been previously described.^48^ First, the built-in rolling ball method was used to subtract background signal from the images. Then, the images were converted into binary images by setting a threshold and the watershed function was used to separate combined puncta. Finally, the “analyze particles” function was used to count instances of expression in the region of interest. The puncta density was calculated as the number of detected puncta divided by the area of the region of interest. Representative images of the analysis were prepared using Adobe Photoshop and Illustrator software. Example images were corrected for brightness and contrast in Adobe Photoshop.

#### In vivo anesthetized electrophysiology

*In vivo* anesthetized electrophysiology of single unit cerebellar Purkinje and nuclear neurons in 6-week-old mice was performed as previously described.^44,49-51^ In short, animals were anesthetized with a ketamine (80 mg/kg) xylazine (16 mg/kg) mixture administered via intraperitoneal injection and maintained with 0.5% isoflurane. Body temperature was maintained throughout the recording session with a heating pad (Kent Scientific, Torrington, CT, USA; #DCT-15). A ~2 mm diameter craniotomy was performed at −6.4 A/P, +/−1.3 M/L to target Purkinje cells. The exposed brain tissue under the craniotomy site was immediately immersed in 0.9% w/v NaCl solution. The recording location was set using a motorized micromanipulator (MP-225; Sutter Instrument Co., Novato, CA, USA). Single unit cell activity was isolated with tungsten electrodes with a resistance of ~5-8 MΩ (Thomas Recording, Giessen, Germany). Electrodes were connected to a preamplifier headstage and bandpass filtered at 0.3–13 kHz (ELC-03XS amplifier, NPI Electronic Instruments, Tamm, Germany). The amplifier output was digitized at 16667 Hz (CED Power 1401, CED, Cambridge, UK). Recordings were made and spike sorted in Spike2 software (CED, Cambridge, UK). Purkinje neurons were identified by the presence of both complex and simple spikes. A minimum of 60 s were analyzed per recording. Spike sorting and analysis were performed by a blinded experimenter. Calculations of the firing properties were made using custom Matlab code (MathWorks, Natick, MA, USA). Firing rate was defined as the count of spikes that occurred within the analysis window (spikes/s). Outliers were identified and removed using the ROUT method (Q = 0.1%) in GraphPad Prism software (GraphPad Software, La Jolla, CA, USA). The number of animals included in the analyses is represented by “n” while the number of cells included in the analyses is represented by “c”.

#### RNA sequencing

Cerebellar, striatal, cortex, hippocampal and brainstem tissue from 10-week-old mice (n=3-4 per genotype) were dissected and frozen in LN2. Total RNA was isolated using the miRNeasy Mini Kit (Qiagen #217004) following manufacturer’s instructions. The RNA was submitted to Genewiz for RNA integrity assessment, library preparation and sequencing on the Illumina HiSeq platform. For each sample, approximately 100 million 150-bp pair-end reads were generated. Raw reads were trimmed before mapping by Trimmomatic-0.36 using the adapter reference TruSeq3-PE.fa:2:30:10.^35^ STAR v2.7.2d was used to align trimmed reads to the Mus musculus genome (GRCm38.p6 M18) and to obtain read counts with default parameters.^33^ Default STAR parameters were used other than –sjdbOverhang 149. The mappabilities for all 79 samples were above 87%.

The read counts were then used for differential gene expression analysis using the DESeq2 package v1.32.0.^34^ Dysregulated genes were called as having an adjusted p-value of < 0.05. Heatmaps were generated using the 1000 genes with lowest P-adjusted values using the pheatmap package v1.0.12. Specifically, the heatmaps show unbiased clustering plotting z-scores for each gene, by normalizing the row to have an average expression of zero and a standard deviation of one. Upset plot was generated using UpSetR package v1.4.0 in R v4.1.1.^32^ KEGG analysis was performed using the clusterProfiler package v4.0.5 in R v4.1.1.^31^ DESeq2 differential gene expression output for all 5 brain regions is included in the Table S2.

#### Nuclear isolation

Nuclei were isolated from frozen 10-week-old cerebella from the following genotypes (*Engrailed1-Cre;Cic*^*fl/fl*^, WT, *Atxn1*^*154Q/2Q*^*, Atxn1*^154Q[V591A;S602D]/2Q^, n = 3-4) using a modified iodixanol gradient.^52^ Briefly, flash frozen tissue was dropped into a 7ml dounce homogenizer containing 5ml buffer HB (0.25M Sucrose, 25mM KCl, 2mM Tricine KOH pH 7.8, 500uM Spermidine) and dounced 10x with loose pestle A and 20x with tight pestle B. Then 320μl of HB-IGEPAL (HB Buffer + 5% IGEPAL CA-630) was added to each homogenizer and dounced 20x more with tight pestle. Each sample was incubated for 10 mins on ice and filtered through a 30μM filter into a conical tube containing 5ml of iodixanol working solution [5 volumes Optiprep (Sigma Aldrich, D15556) + 1 volume Optiprep Diluent (150mM KCl, 30mM MgCl_2_, 120mM Tricine-KOH pH 7.8)] and mixed by tube inversion.

To set up the gradient, 4ml of 40% Iodixanol (3 volumes working solution + 1 volumes HB buffer) was added to a 50ml round bottomed conical tube. Then, 7.5 ml 30% Iodixanol (3 volumes working solution + 2 volumes HB) was slowly overlayed on top, followed by 10ml of the sample containing mixture prepared above. This gradient was spun at 10,000g for 20 mins at 4°C in a hanging bucket centrifuge (Sorvall Lynx 6000) with decel turned off. After centrifugation, nuclei are located at the interface between 30% and 40% iodixanol layers. Iodixanol containing supernatant above the nuclei was slowly discarded with bulb pipette. Approximately 1.5-2ml of the interface containing nuclei were collected and placed into 2ml microcentrifuge tube.

The number of nuclei were quantified by taking 20μl of sample and mixing it with 2μl 0.2mg/ml DAPI diluted in HB buffer. After three-minute incubation at RT, the nuclei were diluted 1:10 in buffer HB and counted on a Countess II with DAPI channel to quantify.

#### CUT&RUN

Cleavage Under Targets & Release Nuclease (CUT&RUN) was performed on nuclei isolated from above following.^17^ As an overview, we performed one nuclear isolation per animal and then split the nuclei into three individual tubes for the three antibodies surveyed (CIC, H3K27ac and IgG). An individual sample will refer to one antibody from a unique animal.

To activate Concanavalin A coated magnetic beads (Bangs Laboratories Inc., BP531) for binding, we incubated 25μl per sample with 3x volumes binding buffer (20mM HEPES-NaOH 7.5, 10mM KCl, 1mM CaCl2, 1mM MnCl2), rotated at RT for 5 minutes, and washed 2x with 1ml binding buffer. All washes are done by placing microcentrifuge tube on magnetic rack and waiting until solution is clear as beads separated from the solution. Following washes, the beads were resuspended in 50ml binding buffer per sample.

After bead activation, 200μl beads were added to 2.0x10^6^ nuclei in the iodixanol mixture from each animal and rotated for 10 minutes at room temperature. Following binding of nuclei to beads, all further processing was done on ice. Bead bound nuclei from each animal was washed 2x with 1ml wash buffer (20mM HEPES NaOH pH 7.5, 150mM NaCl, 500uM Spermidine, and 0.5% Ultrapure BSA [AM2618, Invitrogen] with 1 tablet of Complete Protease Inhibitor Cocktail [Roche 11873580001] per 50ml). After the second wash, 250μl of nuclei bound beads (~500,000 nuclei) in wash buffer were added to individual microcentrifuge tubes corresponding to each antibody.

Supernatant was removed and 250ml of each antibody (CIC 1:2000 [rabbit polyclonal antibody, homemade], IgG 1:100 [rabbit polyclonal, Millipore, 12-370], H3K27Ac 1:100 [Rabbit monoclonal, D5E4 #8173 Cell Signaling]) diluted in antibody buffer (wash buffer + 0.05% Digitonin [11024-24-1, Calbiochem] + 2mM EDTA) was added to the tubes containing nuclei bound during light vortexing (1100rpm). Tubes were then placed at 4°C to rotate overnight. Following rotation, a quick spin on a microcentrifuge was performed to remove liquid from the cap and then washed 2x with 1ml Dig-wash buffer (wash buffer + 0.05% digitonin). Following the second wash, samples were resuspended in 200μl dig wash buffer and transferred to PCR strip tubes. Supernatant was removed and 100μl pAG-MNase [5μl 20x pAG-MNase(15-1016, Epicypher) + 100μl dig wash Buffer] and mixed with gentle flicking. Tubes were placed on nutator for 1hr at 4°C. Following incubation, samples were washed with 200μl dig-wash buffer, transferred to new 1.5ml microcentrifuge tube, and washed 1 additional time in dig-wash buffer.

To initiate cleavage and release of DNA bound fragments, each sample was resuspended in 150μl of dig-wash buffer while gently vortexing. Samples were placed on ice in 4C room for 10 minutes to equilibrate. To start digestion, while in 4°C room, 3μl of 100μM CaCl2 was added to each tube, quickly flicked, and immediately returned to ice. Following 45-minute incubation on ice for digestion, 150μl 2x STOP (340mM NaCl, 20mM EDTA, 4mM EGTA, 0.05% Digitonin, 100μg/ml RNAse A [EN0531, ThermoFisher Scientific], 50 μg/ml Glycogen [10901393001, Roche], and 0.1 ng E. coli spike-in [18-1401, Epicypher] per sample) mixture was added to each sample and then incubated at 37°C for 30 mins to digest RNA and release DNA. E.coli spike-in control was used for normalization of CUT&RUN signal due to differences in library amplification and/or sequencing. Supernatant was transferred to new tube and incubated with 1.5μl 20% SDS and 5μl 10mg/ml Proteinase K while lightly shaking at 50°C for 1hr. DNA was then purified by phenol-chloroform extraction using Maxtract Tubes (129046, Qiagen) and pellet resuspended in 36.5 μl TE Buffer.

#### CUT&RUN library preparation

Library preparation was modified from protocols.IO (https://doi.org/10.17504/protocols.io.bagaibse) utilizing reagents from the NEB Next II DNA Ultra Kit (Cat# E7645S) and NEB Unique Combinatorial Dual index kit (E6442S) with modifications outlined below. Input DNA was quantified with Qubit, and 6ng of CUT&RUN DNA was used as input for the H3K27Ac samples and 25μl of CUT&RUN DNA was used for both CIC and IgG samples. Volume of DNA was brought up to 25ul and 1.5μl End Prep Enzyme Mixture and 3.5 μl Reaction buffer were added and incubated at 20°C for 30 mins and 50°C for 60 mins. After end prep, 15 μl of NEB Next Ultra Ligation Mastermix, 0.5 μl Ligation Enhancer and 1.25 μl of Adapter (1 μM stock adapter for H3K27Ac, 0.5μM stock adapter for CIC and IgG) were added directly to the PCR tube, mixed by pipetting, and incubated for 15 mins at 20°C. Then, 1.5μl of USER Enzyme is added to each tube. Finally, SPRI select beads (B23318, Beckman Coulter) were used at 1.6x ratio to remove excess adapter and eluted in 15μl of TE buffer.

PCR amplification was performed using 13μl of adaptor ligated fragments, 1μl of Unique Combinatorial Dual Index, 1μl of sterile water, and 15μl 2x Q5 Master Mix. 14 cycles of PCR were performed with 10 seconds of denaturation at 98°C and 10 sec of annealing/extension at 65°C. Following PCR amplification, SPRI select beads were used for two-sided size selection; 0.65x right sided selection was performed first followed by 1.2x left sided size selection. Sample was eluted in 15μl TE.

For quality control, each library size distribution was determined by Agilent Tapestation HS DNA 1000 (5067, Agilent Technologies) and concentration was determined by KAPA PCR (07960140001, Roche). Libraries were pooled together at equimolar concentrations and submitted to Genewiz for sequencing. Each library was sequenced for approximately 25 million paired end reads of 150bp in length on a Novaseq 6000 S4 flow cell.

#### CUT&RUN alignment

Our CUT&RUN data analysis pipeline was adapted from CUT&RUN Tools.^53^ Raw Fastq files were appended together using Linux cat function. Adapter sequences were removed from sequence reads using Trimmomatic version 0.36 (2:15:4:4:true LEADING:20 TRAILING:20 SLIDINGWINDOW:4:15 MINLEN:25) from the Truseq3.PE.fa adapter library and kseq.^35,53^ Confirmation of adapter removal and read quality was performed with fastqc (v0.11.8).^54^ Alignment was performed with bowtie2-2.3.4.1 (–dovetail –phred33) to both mm10 (GRCm38 GCA_000001635.2) and the spike-in Ecoli K12 Genomes (GCF_000005845.2_ASM584v2). To isolate small fragments specific to transcription factor binding, fragments under 120 bp in length in CIC and IgG samples using samtools (1.10) as previously described.^53,55^ Bedtools (v2.29.1) was used to process BAM files to BED files, remove blacklist (mm9 blacklist lifted over to mm10 and combined with mm10 blacklist downloaded with CUT&RUNTools,^53^ and to generate bedgraphs.^36^ Each sample was normalized to internal Ecoli spike-in utilizing spike_in_calibration.sh as described previously.^16^ Spike-in normalized bedgraphs from each genotype were merged together using bedtools unionbedg and averaged for summary figures. Both spike-in normalized bedgraphs for each sample and merged bedgraphs were converted to bigwigs using UCSC Bedgraph to Bigwig.

#### CIC peak calling, distribution, and de-novo motif analysis

Replicate BAM files for each factor and genotype were merged using bedtools. Peaks from CIC samples were called by using merged bam file as treatment (WT, *Atxn1*^*154Q/2Q*^, and *Atxn1*^*154Q[V591A;S602D]/2Q*^) compared to control (*Engrailed1-Cre;Cic*^*fl/fl*^) using MACSr (version 1.00, macs3 callpeak parameters “BAMPE”, qvalue = 0.05).^56^ Multiple non-specific peaks were observed in telomeric repeat regions, which were removed by omitting peaks with >50% overlap repeat masked mm10 (RepeatMasker Library db20140131) with bedtools intersect. Distribution of CIC peaks relative to genomic elements was determined using ChIP-seeker plotAnnoPie (v1.28.3).^57^ Association of a peak to gene was determined using bedtools closest and filtering for genes within gene body or within 5kb of promoter. Some genes contain multiple peaks, and the original 2,079 peaks identified correspond to 1,403 unique genes with CIC peaks in the *Atxn1*^*154Q/2Q*^ cerebellum.

De-novo motif analysis was performed with peaks from WT and *Atxn1*^*154Q/2Q*^ animals using the MEME suite (4.11.2).^38^ Briefly, the 50bp sequence flanking each peak summit was isolated from mm10 genome using bedtools getfasta and de-novo motif analysis using MEME-ChIP, MEME output.^58^ Identified de-novo motif was compared to similarly reprocessed data CIC ChIP-seq peaks using TomTom.^15,20^ CIC CUT&RUN peaks are included in Table S6.

#### Visualization and differential quantification of CUT&RUN signal

Integrated Genome Viewer (IGV) v2.11.1 was used to examine spike-in normalized signal tracks at individual loci.^30^ Target loci for this analysis was determined as follows: For signal at all CIC peaks, we extracted genomic coordinates for summits andincluded 50bp on either side of the summit. Additionally, we subset *Atxn1*^*154Q/2Q*^ peaks within 5kb of DEGs from *Atxn1*^*154Q/2Q*^ RNA-seq (padj <0.05). Similarly, we subset coordinates for all gene bodies of *Atxn1*^*154Q/2Q*^ DEGs (padj <0.05) for examination of H3K27Ac signal.

To globally visualize CUT&RUN signal at above loci, we used deeptools plotProfile and plotHeatmap functions from spike-in normalized bigwig files (version 3.5.1).^39^ Quantification of CIC binding at CIC peaks and H3K27Ac binding within gene bodies of DEGs were generated using bedtools multicov. Briefly, we utilized bed files representing CIC peaks or gene bodies and bedtools multicov was used to generate a matrix of raw reads from each individual replicate at each locus. The number of aligned spike-in reads per sample was added as a row. This was imported into DeSeq2 for downstream processing.

Differential binding was calculated between WT and *Atxn1*^*154Q/2Q*^ and *Atxn1*^*154Q[V591A;S602D]/2Q*^ genotypes using DESeq2 package (v1.32.0).^34^ Counts were normalized to the spike-in E coli control.

#### Motif bootstrapping

FIMO from MEME Suite version 4.11.2 was utilized to identify the occurrence of each motif (CIC this paper, ZKSCAN1 and RFX1 from JASPAR) within 1 kb upstream of the transcriptional start site (TSS).^59^ For peaks, bedtools closest was utilized to pair a peak with a gene within 5kb of TSS and analyzed in similar fashion. The frequency of differentially expressed genes for each RNA-seq dataset (padj < 0.05) containing a motif or peak was calculated by (DEGs with motif or peak/Total DEGs per RNA-Seq Dataset) and represented as a black dot. To examine significance of the observed frequency relative to random chance, we calculated the frequency of the motif or peak within a random set of the same number of non-differentially expressed genes (padj > 0.05) and randomly sampled 10,000 repetitions with each simulated repetition represented as a colored dot (salmon or blue depending on genotype). P value was then computed as (r+1)/(n+1), where r is the number of repetitions where percent motifs in selected non-DEGs is greater than the percent of motifs or peaks in DEGs experimentally determined and n is the total number of repetitions.^60,61^ Plots are made in R version 4.11 with package ggplot version 3.3.5.^62^ CIC, RFX1 and ZSKCAN1 motif analyses are included in Tables S3, S4, and S5, respectively.

#### ATXN1 immunoprecipitation

Tissue from 4-week-old mice were lysed in 1mL NEMT Buffer (50 mM Tris pH 7.5, 0.5% Np-40, 150 mM NaCl, 1mM EDTA) supplemented with fresh 1X protease inhibitor (Roche) and 1X phosphatase inhibitor (Sigma) using a Dounce homogenizer (50x with 2mL size homogenizer, using Pestle B). Lysate was incubated on ice for 20 mins and then centrifuged at max speed for 20 mins at 4°C. Pierce BCA protein kit (Cat # 23227) was utilized to quantify protein and equal amounts of protein were used for each IP. 15μl of Protein G Dynabeads were washed 3x with 500μl of 1xPBS and then incubated at RT for 40 mins with 600μl 5% BSA in 1x PBS with either 1μl of normal mouse IgG (Millipore, #12-371) or 5 μl of mouse anti-ATXN1 (in house).^63^ Beads were washed 2x with 500μl NEMT on ice, and then 300μl of the lysate was loaded on the washed beads and incubated at 4°C for 45 mins. Beads were washed 4x with 500μl NEMT on ice and in second wash, beads were moved to a new tube. After final wash, loading buffer was added and beads were boiled for 10 mins at 95°C. After elution, beads were removed and samples were ran on NuPAGE 4-12% Bis-Tris 1.5mm 15-well gels (NP0336BOX).

For mass spectrometry, a preclearing step was added and lysate was incubated with Protein G Dynabeads (not coated with additional antibody) for 30 mins on a rotator at 4°C. Upon removal of lysate, these beads were used as the negative mass spectrometry control. The lysate was then added to antibody coated Protein G Dynabeads and the immunoprecipitation was carried out as described.

#### Mass spectrometry

The affinity purified protein and its interacting proteins were digested on beads. The beads were washed with cold PBS twice and resuspended with 50 μl of 20 mM Ammonium bicarbonate (pH8.0), 2mM CaCl_2_ and 500 ng of MS- grade trypsin was added and digested for overnight at 37°C. The digestion was stopped by adding 5 ul of 10% formic acid. The digested peptide was removed and enriched by in-housed STAGE tip^64^ column with 2 mg of C18 beads (3 μm, Dr. Maisch GmbH, Germany) then vacuum dried. Resuspended peptides were subjected to a nanoLC-1000 (Thermo Scientific) coupled to Orbitrap Fusion Lumos^™^ mass spectrometer (Thermo Scientific) with ESI source. The peptides were loaded onto an in-house Reprosil-Pur Basic C18 (1.9 μm, Dr. Maisch GmbH, Germany) trap column (2 cm length, 100 μm i.d.) and separated by 5 cm column (150 μm i.d.) with a 75 min gradient of 2-28 % of acetonitrile/0.1% formic acid at a flow rate of 800 nl/min. The data acquisition was made in data dependent analysis mode (DDA) for unbiased peptide detection. The precursor MS spectrum was scanned at 300-1400 m/z, 120k resolution at 400 m/z, 5x105 AGC target (50 ms maximum injection time) by Orbitrap. Top 30 scan was applied to selected MS1 signal and filtered by Quadrupole (2 m/z isolation window, 15 second exclusion time with +/− 7ppm mass tolerance), fragmented by Higher-energy C-trap dissociation (HCD) and detected by Ion trap with rapid scan rate (5x103 AGC target, and 35 msec of maximum injection time). Obtained spectra were searched against the target-decoy mouse RefSeq database (release Dec. 2020, 28,456 protein sequence) in Proteome Discoverer 2.1 interface (PD 2.1, Thermo Fisher) with the Mascot algorithm (Mascot 2.4, Matrix Science). Dynamic modifications of the acetylation of protein N-terminus and oxidation of methionine were allowed. The precursor mass tolerance was confined within 20 ppm with fragment mass tolerance of 0.5 Da and a maximum of two missed cleavages was allowed. Assigned peptides were filtered with 1% false discovery rate (FDR) using percolator validation based on q-value. Calculated area under curve of peptides was used to calculate iBAQ for protein abundance using in housed software.^65^

Significant proteins were identified by an absolute log_2_ fold change ≥ 4 and a p-value < 0.05 when compared to the preclear control. The list of mouse transcription factors came from AnimalTFDB3.0.^66^ ATXN1 IP-MS data is included in Table S9.

#### Analysis of public ChIP-seq data

Public mouse ChIP-seq data for RFX1 and ZKSCAN1 was downloaded from http://cistrome.org/db/#/.^67^ RFX1 ChIP-seq was derived from neural precursor cells (NPCs)^24^ and ZKSCAN1 ChIP-seq was derived from erythroid progenitor cells.^25^ After downloading BED peak files of each transcription factor, association of a peak to gene was determined using bedtools closest and filtering for genes within gene body or within 5kb of promoter. Pie charts were generated by quantifying how many *Atxn1*^*154Q/2Q*^ DEGs contained a peak for just one transcription factor (CIC, RFX1 or ZKSCAN1), peaks from multiple transcription factors (TFs; CIC and RFX1, CIC and ZKSCAN1, RFX1 and ZKSCAN1, or CIC, RFX1 and ZKSCAN1) and those DEGs which contain no TF peaks. RFX1 and ZKSCAN1 ChIP-seq peaks are included in Tables S7 and S8, respectively.

#### iPSC Differentiation into iNeurons

Fibroblast conversion into iPSCs was previously described,^9^ but briefly, fibroblasts were derived from skin biopsies collected from individuals with SCA1 and their unaffected siblings after obtaining written, informed content as approved by the institutional Review Board of the Human Subjects Committee at the University of Minnesota. The age and gender of these individuals has not been reported to respect the wishes of anonymity of this kindred. iPS cells were reprogrammed from fibroblasts.^68^ Fibroblast cells were reprogramed with CytoTune-iPS 2.0 kit (Invitrogen) follow the manufacturer’s instructions. iPSCs were plated in a 6-well low binding plate and infected with a lentivirus packaged with rtTA with gentamicin selection, with polybrene added as a transfection reagent. Cells were shaken overnight in an incubator to form spheres, and after 24 hours transferred into 4 wells of a 6-well 2x Cultrex plate. One hour after transfer, media was changed to remove excess virus and polybrene. The following day, gentamicin selection was started with daily media changes until uninfected control cells were absent. The cells were then infected in the same method with lentivirus packaged with doxycycline inducible NGN2-EGFP with puromycin selection. After infection, one well of the transferred cells was inducted with doxycycline to check infection efficiency. Neurons were differentiated as described^69^ with 75,000 cells/well in a Matrigel coated 24-well plate. Neurons matured for 21 days prior to RNA isolation. RNA isolation and cDNA synthesis described above. Genes selected to test for qRT-PCR in the iNeurons met the criteria of having an adjusted p-value < 0.05 in the mouse RNA-seq data, a predicted peak of only one transcription factor, and were expressed in iNeurons according to https://ineuronrnaseq.shinyapps.io/rnaseq_app/. Genes that met these criteria were then ranked by log2FC and the highest were selected for testing. 30% of the genes screened reproduced in the human iNeurons. Genes that reproduced in the iNeurons were selected as representative genes to show for the mouse RNA-seq data in Figures 4G-4I. Primer sequences for qRT-PCR are included in Table S1.

#### Statistical analysis

Experimental analysis was performed in a blinded manner when possible. Statistical tests were performed in accordance with the experimental design. Simple comparisons used Student’s t-test, whereas multi-group comparisons used one or two-way ANOVAs. Nonparametric data used Mann-Whitney tests. Survival analysis used Log-rank test. In each case, *, **, ***, **** and ns denote p<0.05, p<0.01, p<0.001, p<0.0001 and p>0.05, respectively.

## Supplementary Material

MMC7

MMC8

MMC9

MMC6

MMC5

MMC4

MMC2

MMC10

MMC1

MMC3

## Figures and Tables

**Table:** KEY RESOURCES TABLE

REAGENT or RESOURCE	SOURCE	IDENTIFIER
Antibodies		
Rabbit Polyclonal Anti-ATXN1	In house (Servadio et al.^27^)	11750, RRID:AB_2721278
Rabbit Polyclonal Anti-CIC	In house (Lam et al.^7^)	RRID:AB_2721281
Mouse Monoclonal Anti-GAPDH	Jackson ImmunoResearch	Cat # #715-035-150, RRID:AB_2107426
Rabbit Monoclonal Anti-H3K27ac	Cell Signaling	Cat# 8173,RRID:AB_10949503
Rabbit Polyclonal Anti-CAR8	Proteintech	Cat# 12391-1-AP, RRID:AB_2066277
Rabbit Polyclonal Anti-Mouse IP3R1	Thermo Fisher Scientific	Cat# PA1-901, RRID:AB_2129984
Rabbit Polyclonal Anti-RFX1	Bethyl	Cat# A303-043A, RRID:AB_10754499
Rabbit Polyclonal Anti-ZBTB5	Atlas Antibodies	Cat# HPA021521, RRID:AB_2670736
Critical commercial assays		
miRNeasy Mini Kit RNA Isolation	Qiagen	Cat# 217004
Next II DNA Ultra Kit	New England BioLabs	Cat# E7645S
Unique Combinatorial Dual index kit	New England BioLabs	Cat# E6442S
Deposited data		
WT, *Atxn1^154Q/2Q^* and *Atxn1^154Q[V591A;S602D]/2Q^* RNA-seq	This Paper	GEO: GSE218283
CIC & H3K27ac CUT&RUN	This Paper	GEO: GSE218301
Entire RNA-seq & CUT&RUN Dataset	This Paper	GEO: GSE218302
WT and *Engrailed-1-Cre; Cic^flox/flox^* RNA-seq	Rousseaux et al.^9^	GEO: GSE108254
RFX1 ChIP-seq	Creyghton et al.^24^	GEO: GSM594584
ZKSCAN1 ChIP-seq	Yue et al., 2014^25^	GEO: GSM1003779
Experimental models: Cell lines		
The generation of iPSC-derived neurons from an SCA1 kindred is described in detail in the STAR Methods	This paper	Available upon request
Experimental models: Organisms/strains		
Mouse: C57BL6/J	The Jackson Laboratory	RRID:IMSR_JAX:005304
Mouse: *Atxn1*^2Q/−^ (over null)	Matilla et al.^28^	RRID:IMSR_JAX:029025
Mouse: *Atxn1^154Q/2Q^*	In House (Watase et al.^10^)	RRID:IMSR_JAX:005601
Mouse: *Atxn1^154Q[V591A;S602D]/2Q^*	This Paper	JAX Stock # 037673
Mouse: *Atxn1^2Q[V591A;S602D]/2Q^*	This Paper	JAX Stock # 037674
Mouse: *Engrailed-1-Cre*	Kimmel et al., 2000^29^	RRID:IMSR_JAX:007917
Mouse: *Cic^flox/flox^*	Lu et al.^18^	RRID:IMSR_JAX:030555
Oligonucleotides		
ssODN for V591A;S602D mice: CAAACTGTATCACGGCCACCCCGGGGCTGTGGCTCTCCTCGATTCTCTCCACAGTACTGGAGTCGATCTTGAGGTCATTGCTAATCTCTGCAtcCTGGATGAAATCtTCtGTtTTtAGaTCtTCggCCTTCTTCAGCTCCCCGTTGGCCAGCTGGATGATGGA	This paper/IDT	NA
crRNA for V591A;S602D mice: GGTGGAGGACCTGAAGACGG	This paper/IDT	NA
tracrRNA for V591A;S602D mice	IDT	Cat# 1072532
qPCR Primers	This paper/Sigma	See Table S1
Genotyping Primers	This paper/Sigma	See STAR Methods Details
Software and algorithms		
IGV v2.11.1	Robinson et al.^30^	https://software.broadinstitute.org/software/igv/
Clusterprofiler v4.0.5	Yu et al.^31^	https://bioconductor.org/packages/release/bioc/html/clusterProfiler.html
UpsetR v1.4.0	Conway et al.^32^	https://upset.app/
STAR v2.7.2d	Dobin et al.^33^	https://github.com/alexdobin/STAR
DESeq2 v1.32.0	Love et al.^34^	https://bioconductor.org/packages/release/bioc/html/DESeq2.html
Trimmomatic v0.36	Bolger et al.^35^	http://www.usadellab.org/cms/?page=trimmomatic
Bedtools v2.29.1	Quinlan et al.^36^	https://bedtools.readthedocs.io/en/latest/
MACSr v1.00	Hu, 2022^37^	https://www.bioconductor.org/packages/release/bioc/html/MACSr.html
MEME suite v4.11.2	Bailey et al.^38^	https://meme-suite.org/meme/
Deeptools v3.5.1	Ramírez et al.^39^	https://deeptools.readthedocs.io/en/develop/

## Highlights

- ATXN1-CIC complex drives SCA1 Purkinje cell loss but minimally affects other cells
- CIC DNA binding is enhanced in SCA1 and normalized by disrupting the ATXN1-CIC complex
- ATXN1 interacts with additional transcription factors RFX1, ZBTB5, and ZKSCAN1
- Targets of multiple ATXN1 interactors are altered in SCA1 mice and patient cells
