# Disruption of the ATXN1-CIC complex reveals the role of additional nuclear ATXN1 interactors in spinocerebellar ataxia type 1

**DOI:** 10.1016/j.neuron.2023.02.030

**Published:** 2023-03-15

**Authors:** Stephanie L. Coffin, Mark A. Durham, Larissa Nitschke, Eder Xhako, Amanda M. Brown, Jean-Pierre Revelli, Esmeralda Villavicencio Gonzalez, Tao Lin, Hillary P. Handler, Yanwan Dai, Alexander J. Trostle, Ying-Wooi Wan, Zhandong Liu, Roy V. Sillitoe, Harry T. Orr, Huda Y. Zoghbi

In the originally published paper, there was one error in the STAR Methods section “Generation of *Atxn1*^*154Q[V591A;S602D]/2Q*^ and *Atxn1*^*2Q[V591A;S602D]/2Q*^ mouse models.” When describing the mouse amino acid locations for the two mutations made in the AXH domain of ATXN1, we erroneously stated that human V591 and S602 correspond to mouse V620 and S631, respectively. The corrected text should read “Please note that human V591 and S602 correspond to mouse V566 and S577, respectively.” This has now been updated online. The authors apologize for the error.

