# Supplementary material for: Disruption of the ATXN1-CIC complex reveals the role of additional nuclear ATXN1 interactors in spinocerebellar ataxia type 1
Source: Neuron. Author manuscript; Available in PMC 2023 Feb 25. (PMC9957872; doi:10.1016/j.neuron.2022.11.016)
Supplement: MMC1 [file NIHMS1859677-supplement-MMC1.pdf]

Neuron, Volume *111*

Supplemental information

Disruption of the ATXN1-CIC complex reveals the role of additional nuclear ATXN1 interactors in spinocerebellar ataxia type 1

Stephanie L. Coffin, Mark A. Durham, Larissa Nitschke, Eder Xhako, Amanda M. Brown, Jean-Pierre Revelli, Esmeralda Villavicencio Gonzalez, Tao Lin, Hillary P. Handler, Yanwan Dai, Alexander J. Trostle, Ying-Wooi Wan, Zhandong Liu, Roy V. Sillitoe, Harry T. Orr, and Huda Y. Zoghbi

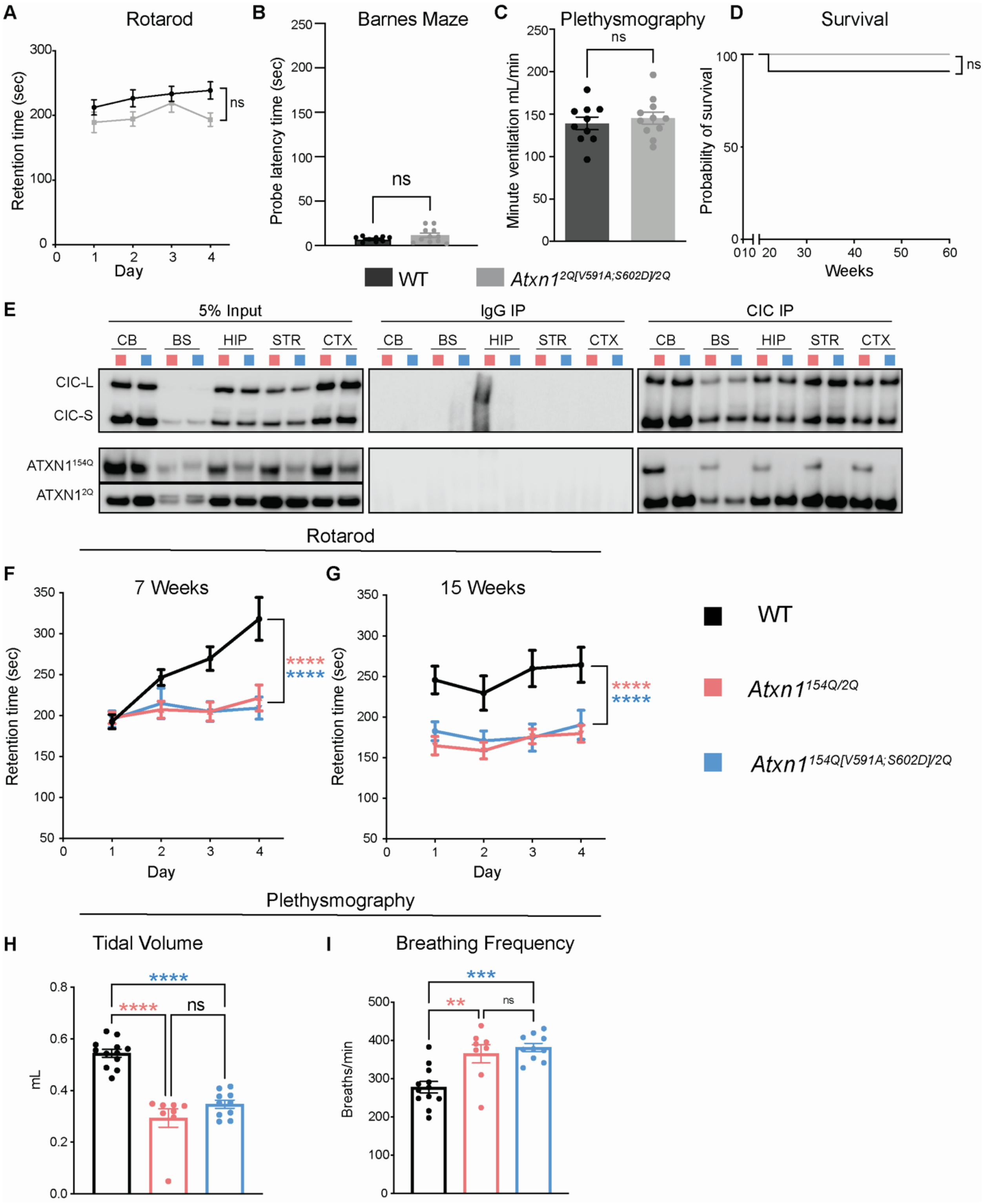

Supplemental Figure 1. Global loss of ATXN1^{154Q}-CIC binding partially improves SCA1 phenotypes. Related to Figure 1 and 2. A) Rotarod assay at 7-weeks of age in WT and *Atxn1$^{2Q[V591A;S602D]/2Q}$* mice. **B)** Barnes maze at 14-weeks of age in WT and *Atxn1$^{2Q[V591A;S602D]/2Q}$* mice. **C)** Minute ventilation as measured via

plethysmography at 40-weeks of age in WT and *Atxn1*$^{2Q[V591A;S602D]/2Q}$ mice. **D)** Survival analysis of WT and *Atxn1*$^{2Q[V591A;S602D]/2Q}$ mice. **E)** Immunoprecipitation (IP) of CIC and representative western blot for ATXN1 and CIC in *Atxn1*$^{154Q/2Q}$ and *Atxn1*$^{154Q[V591A;S602D]/2Q}$ mice in cerebellum (CB), brainstem (BS), hippocampus (HIP), striatum (STR), and cortex (CTX) at 4-weeks of age. Rotarod assay in **F)** 7-week-old and **G)** 15-week-old WT, *Atxn1*$^{154Q/2Q}$ and *Atxn1*$^{154Q[V591A;S602D]/2Q}$ mice. Plethysmography measuring **H)** tidal volume and **I)** breathing frequency in WT, *Atxn1*$^{154Q/2Q}$ and *Atxn1*$^{154Q[V591A;S602D]/2Q}$ mice at 40-weeks of age. For each assay, a minimum of 8 mice were used. Two-way ANOVAs with Tukey's multiple comparisons were used for A, F and G. T-tests were used for B and C. Mantel-Cox log-rank was used for D. One-way ANOVAs with Tukey's multiple comparisons were used for H and I. In each case, *, **, ***, **** and ns denote $p<0.05$, $p<0.01$, $p<0.001$, $p<0.0001$ and $p>0.05$, respectively. All data are represented as means ± SEM.

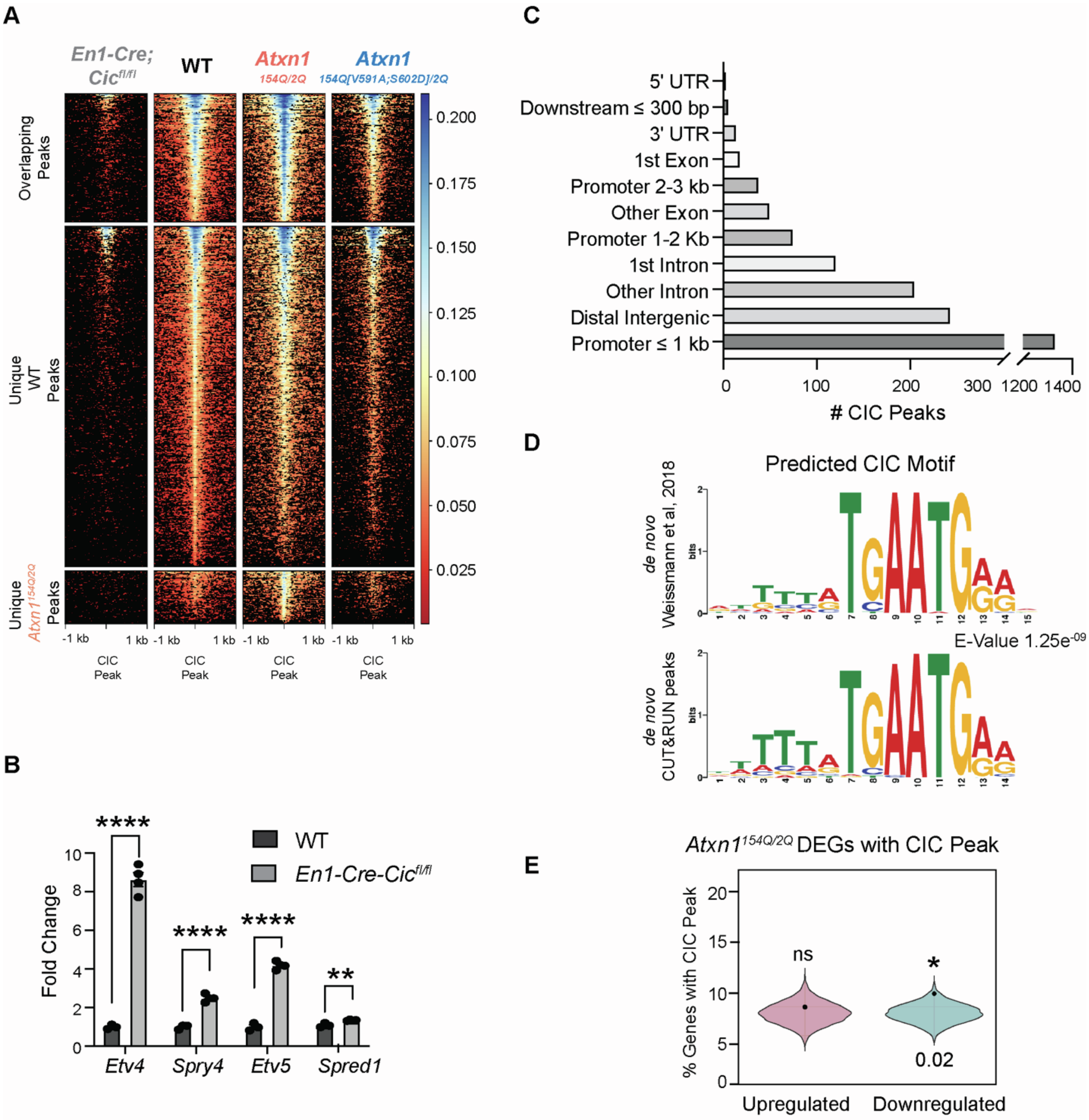

Supplemental Figure 2. CIC peaks identify significant consensus motif and CIC as a transcriptional repressor in SCA1. Related to Figure 3. A) Heatmap of CIC signal at unique and overlapping peaks for WT and *Atxn1*$^{154Q/2Q}$. **B)** Fold change of normalized gene counts in WT and *En1-Cre;Cic*$^{fl/fl}$ mice for CIC target genes. RNA-sequencing data from Rousseaux et al., 2018. **C)** Distribution of *Atxn1*$^{154Q/2Q}$ CIC peaks by gene element. **D)** TOMTOM analysis comparing *de novo* CIC motifs generated from CUT&RUN data here and previously published CHIP-seq data from Weissman et al, 2018. Two motifs and the corresponding E-value of the strength of the similarity depicted. **E)** Violin plots of CIC peak enrichment in *Atxn1*$^{154Q/2Q}$ up and down-regulated DEGs compared to random genes, ran over 10,000 experiments. T-Tests were used in B. In each case, *, **, ***, **** and ns denote p<0.05, p<0.01, p<0.001, p<0.0001 and p>0.05, respectively.

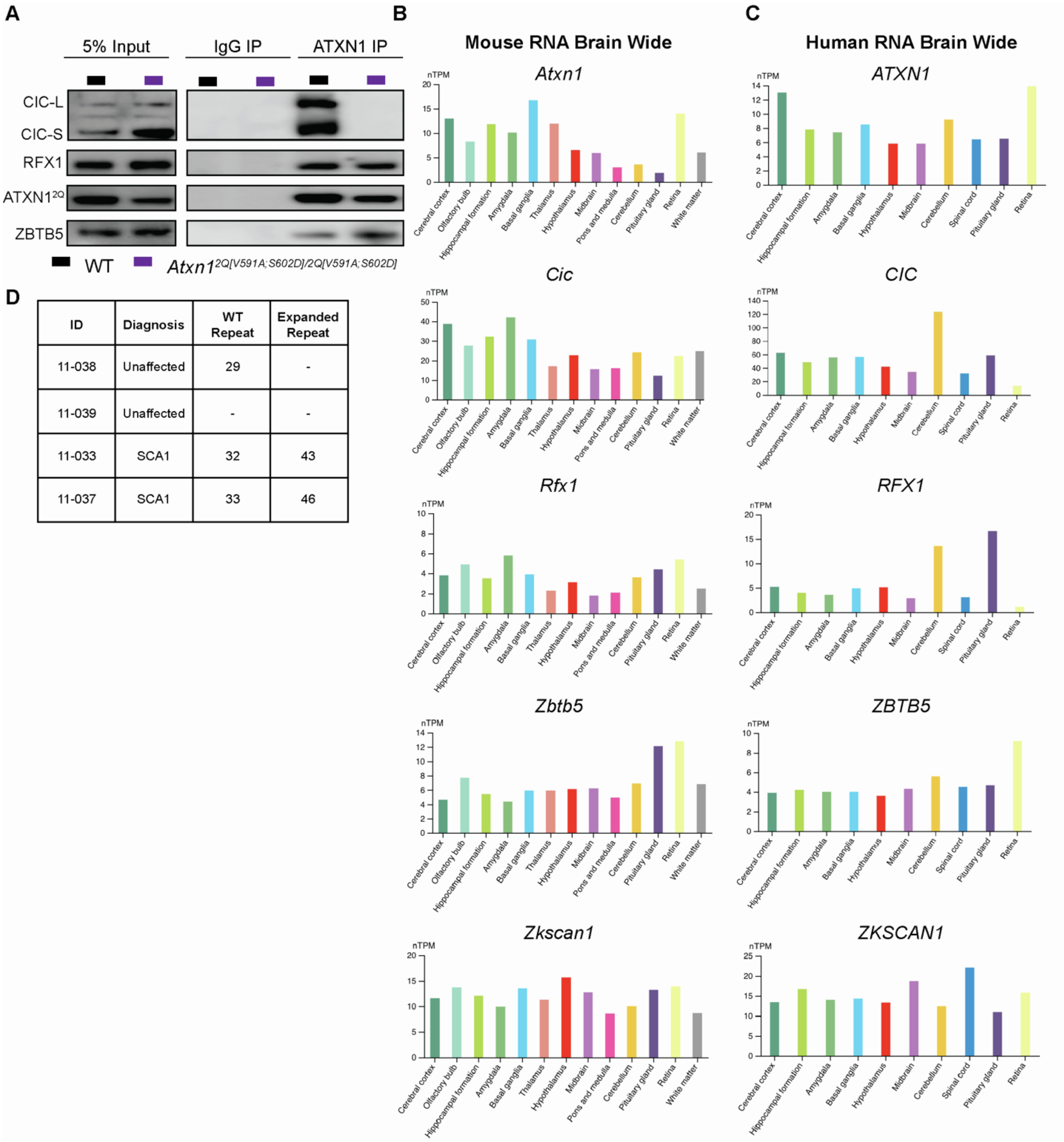

ID	Diagnosis	WT Repeat	Expanded Repeat
11-038	Unaffected	29	-
11-039	Unaffected	-	-
11-033	SCA1	32	43
11-037	SCA1	33	46

Supplemental Figure 3. Novel ATXN1 interactors are expressed brain wide. Related to Figure 4. A) Representative western blot showing the pulldown of ATXN1, CIC, RFX1 and ZBTB5 upon immunoprecipitation (IP) of ATXN1 in cerebella of WT and *Atxn1*$^{2Q[V591A;S602D]/2Q[V591A;S602D]}$ mice at 4-weeks of age. **B)** Human protein atlas RNA expression of mouse *Atxn1*, *Cic*, *Rfx1*, *Zbtb5*, and *Zkscan1* brain wide. **C)** GTEx RNA expression of human *ATXN1*, *CIC*, *RFX1*, *ZBTB5*, and *ZKSCAN1* brain wide. **D)** CAG repeat information of unaffected sibling and SCA1 patient samples collected for iPSC and iNeuron differentiation.
